# Microbiota and Nutrient Portraits of European Roe Deer (*Capreolus capreolus*) Rumen Contents in Characteristic Southern German Habitats

**DOI:** 10.1007/s00248-023-02308-5

**Published:** 2023-10-24

**Authors:** Sarah-Alica Dahl, Jana Seifert, Amélia Camarinha-Silva, Yu-Chieh Cheng, Angélica Hernández-Arriaga, Martina Hudler, Wilhelm Windisch, Andreas König

**Affiliations:** 1 Wildlife Biology and Management Unit, Chair of Animal Nutrition and Metabolism, Technical University of Munich, Hans-Carl-von-Carlowitz-Platz 2, 85354 Freising, Germany; 2 HoLMiR – Hohenheim Center for Livestock Microbiome Research, University of Hohenheim, Leonore-Blosser-Reisen-Weg 3, 70599 Stuttgart, Germany; 3 Institute of Animal Science, University of Hohenheim, Emil-Wolff-Str. 10, 70599 Stuttgart, Germany; 4 Game Management and Wildlife Management, Weihenstephan-Triesdorf University of Applied Sciences, Hans-Carl-von-Carlowitz-Platz 3, 85354 Freising, Germany; 5 TUM School of Life Sciences, Technical University of Munich, Liesel-Beckmann-Straße 2, 85354 Freising, Germany

**Keywords:** Roe deer, *Capreolus capreolus*, Microbiota, Rumen content, Bacteria, Habitat

## Abstract

Roe deer (*Capreolus capreolus*) are found in various habitats, from pure forest cultures to agricultural areas and mountains. In adapting to the geographically and seasonally differentiating food supply, they depend, above all, on an adapted microbiome. However, knowledge about the microbiome of wild ruminants still needs to be improved. There are only a few publications for individual species with a low number of samples. This study aims to identify a core microbiota for Bavarian roe deer and present nutrient and microbiota portraits of the individual habitat types. This study investigated the roe deer’s rumen (reticulorumen) content from seven different characteristic Bavarian habitat types. The focus was on the composition of nutrients, fermentation products, and the rumen bacterial community. A total of 311 roe deer samples were analysed, with the most even possible distribution per habitat, season, age class, and gender. Significant differences in nutrient concentrations and microbial composition were identified for the factors habitat, season, and age class. The highest crude protein content (plant protein and microbial) in the rumen was determined in the purely agricultural habitat (AG), the highest value of non-fibre carbohydrates in the alpine mountain forest, and the highest fibre content (neutral detergent fibre, NDF) in the pine forest habitat. Maximum values for fibre content go up to 70% NDF. The proportion of metabolites (ammonia, lactate, total volatile fatty acids) was highest in the Agriculture-Beech-Forest habitat (ABF). Correlations can be identified between adaptations in the microbiota and specific nutrient concentrations, as well as in strong fluctuations in ingested forage. In addition, a core bacterial community comprising five genera could be identified across all habitats, up to 44% of total relative abundance. As with all wild ruminants, many microbial genera remain largely unclassified at various taxonomic levels. This study provides a more in-depth insight into the diversity and complexity of the roe deer rumen microbiota. It highlights the key microorganisms responsible for converting naturally available nutrients of different botanical origins.

**Supplementary Information:**

The online version contains supplementary material available at 10.1007/s00248-023-02308-5.

## Introduction

The roe deer (*Capreolus capreolus*) has evolved in recent decades from a typical forest edge and scrub habitat dweller to an inhabitant of diverse habitat types [1, 2]. As a typical synanthropic species, it increasingly uses agricultural habitats, where the energy supply is sometimes even higher than in pure forest areas and to which it is optimally adapted to browse [3, 4]. Nevertheless, various types of forest, alpine areas, or grassland farming are also used by roe deer. The diverse habitats give the animals different forage offers, both seasonally and regionally, because of the plant availability and nutrient composition [3, 5, 6].

The essential tool for adaptation to these conditions is the ruminal microbiome of the roe deer, which is the key for the ruminant to access energy and nutrients from the plants. The rumen forms the first anaerobic digestive chamber and is inhabited by a diverse microbial community. The bacteria and fungi living in it provide the required carbohydrate-active enzymes for the hydrolysation of complex polysaccharides. The following microbial fermentation processes generate volatile fatty acids (VFAs), which the host uses as a primary energy source [7, 8]. In addition to active fermentation, the microbiome also serves as an essential non-vegetable protein source for the host forming the central part of the protein that the host can digest. Most of the protein supplied through food intake is converted directly into ammonia and is then used to build microbial protein [9].

The roe deer is a selector [4] and can efficiently break down and utilise high fibre content in the ingested forage. Thanks to microbial plasticity, they are largely resistant to changes in diet composition [3]. It shows snacking habits and consumes many different plants per browsing period. It prefers young shoots, buds, shrub fruits, tree fruits, and herbs, if available [10]. However, grass and woody plant parts are also integral to the diet. Over 300 plant species are known to be ingested by the deer [6, 11, 12], which adapts to the seasonal and local supply. The roe deer takes its forage, distributed over the day, in an average of 8–11 browsing periods [11, 12]. The passage rate is, therefore, much faster than for typical grazers [11]. In times of lower or less diverse food availability, however, it can also adapt to even more fibre-rich food, which has to stay longer in the rumen. One adaptation mechanism is, for example, the increased filling of the rumen per browsing period in winter and the resulting longer digestion and rumination times [4].

The degree of tolerance to the fibre content in selectors (or browsers) is still highly discussed. Although there is sufficient evidence to the contrary, it is still often said that selectors can only utilise small amounts of fibre and depend on forage with a very high protein and energy content, even in scientific circles. Browsing areas are often evaluated only based on the protein content of the plants. It was also assumed for a long time that selectors such as roe deer had hardly any cellulose-utilising bacteria in their rumen [13]. But fortunately, the point of view has slowly changed. The rigid picture of categorising ruminants as roughage eaters, intermediate types, and concentrate selectors [11] is being replaced by the knowledge that the transitions between the individual feeding types are fluid. Therefore, we can speak of a “browser-grazer continuum” [14]. However, the field of wildlife nutrition continues to offer much potential for further research.

The extent to which ruminants adapt smoothly to nutritional changes is known mainly from numerous dairy cow, beef cattle, and sheep studies [15–17]. Literature on wild ruminants is rather limited and often based on minimum sample size [18]. In addition, the correct identification and functional assignment of individual bacterial genera of the microbiota is not trivial, as many species still need to be isolated and described [19]. So, there is still an enormous gap in knowledge in this field.

This study aims to identify the core microbiota of Bavarian roe deer and promote a basis for future comparability of wildlife studies. The second aim was to describe the variations of the rumen microbiota and its fermentation products of free-living roe deer from seven different characteristic Bavarian habitat types throughout the year. When communicating with parties involved in wildlife management (such as foresters, hunters, or farmers), it is often argued that results from other habitats are not comparable to their local situation. To avoid this, the most characteristic and extreme Bavarian habitat communities from different growth areas were selected in this study to present a comprehensive picture.

We hypothesise that a dynamic adaptation of the nutritional conditions is reflected by changes in the rumen microbiota depending on the respective ecological habitat.

## Materials and Methods

### Sample Material and Study Areas

Within the framework of this study, roe deer samples were collected from seven different project areas (Table 1). The seven areas represent the most typical habitat types in Bavaria (Germany). They cover habitats with extreme conditions (in terms of climate or forage availability), such as pine forests, mountains, or pure agricultural landscapes, as well as typical forest communities and mixed forms with agricultural and forest components (like habitat ABF). They also show their primary occurrence at the selected sample sites, and each harbours large roe deer populations. Besides differences in altitude, geological aspects, and local climatic parameters (Table S3), habitats differ primarily in the composition of the plant community and, thus, the available forage for wildlife. For more habitat information, see Table 1 and Table S3 (suppl. information). Five habitats are managed by the Bayerische Staatsforsten AöR (Bavarian Forestry Authority, a public-law institution), one by the University of Würzburg, and one privately. The areas within the habitats were selected with regard to accessibility, on-site support, and the absence of anthropogenic feeding. Offered feed is only found in the form of apple pomace for attraction during hunting (autumn/winter).

**Table 1:**
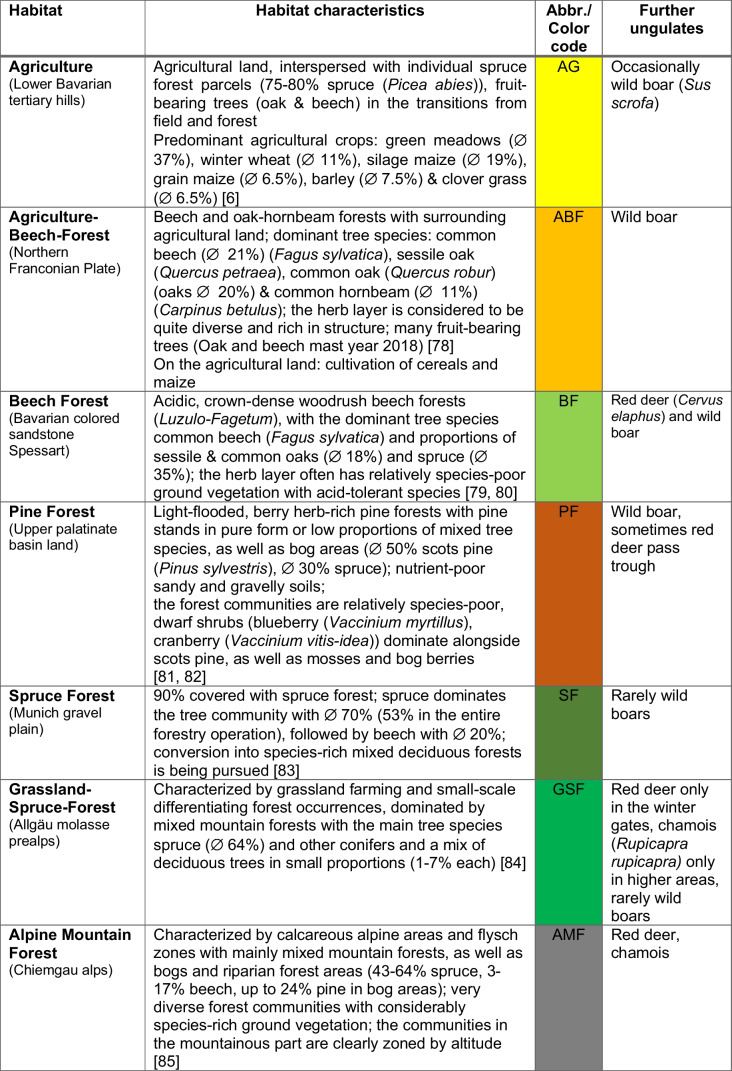
List of sample areas investigated, including habitat characteristics and further ungulates

All roe deer samples were obtained during regular hunting activities and shooting schedules. To ensure continuous sampling throughout the year, closed seasons, variable by forestry, were suspended for males and subadult females between November and May. After the culling, the entire rumen (reticulorumen) with its contents and other organs were removed and packed in plastic bags. The samples were frozen to at least −18C° as soon as possible after collection. In contrast to experiments with domestic ruminants under controlled conditions, sampling is impossible within a few minutes in the context of hunting activity. On average, 30–60 min can elapse between shooting, organ removal, and freezing. The samples were almost exclusively obtained during individual hunts, which allows for faster sampling than during social hunts. This is an unavoidable bias in wildlife sampling.

Furthermore, the condition and constitution data of each animal were recorded. The age was determined based on tooth development (in young animals) or the tooth section method [20] in older animals (from the age of 2) and was divided into age classes (juvenile, subadult, adult) (distribution of frequencies in Table S2). For the microbiome studies, 311 roe deer from all ages and both gender were sampled. Samples for the spruce forest (SF) and agriculture (AG) habitats were collected in 2011–2014 as part of the preliminary study [6], and all other samples between 2017 and 2019 as part of the ongoing. Information regarding vegetation surveys and botanical rumen content analyses are given in Supplementary Information (Fig. S1) and were previously published [5]. Additional information on the climate data of the individual habitats is shown in Table S3.

The samples were thawed for further processing in the laboratory. The rumen was separated and opened, and the complete content (solid and liquid content) was homogenised by stirring. Approximately 500ml of homogenised content was removed and refrozen. For the separation of 1.5ml for DNA extraction, the content was later thawed and homogenised again. After the separation of material for DNA extraction, 10–15ml of supernatant rumen juice was filled or, if necessary, passed through filter paper to analyse metabolites and refrozen.

### Analyses of Crude Nutrients and Fermentation Products

The thawed rumen contents (ca. 500ml) were centrifuged (4400 × g, 15 min, 21°C), and the precipitate was freeze-dried and ground to 1mm grain size.

The crude nutrients [%/DM] (crude protein (CP), total lipids (TL), ash, acid detergent fibre (ADF (ADFom, after ashing)), neutral detergent fibre (NDF, also referred to as “total fibres” in the text (aNDFom, after ashing and amylase treatment)), and acid detergent lignin (ADL/lignin) were analysed, and the fibre fractions were calculated using standard feed analysis procedures. Hemicellulose results from the difference between NDF and ADF, and cellulose from the difference between ADF and lignin. In addition, the crude fibre content (CF) was analysed for comparison with older studies. The proportion of non-fibre-carbohydrates (NFC) was calculated from the difference between the dry masses and the other crude nutrients (Weender and VanSoest analysis; Methods 3.1, 4.1.1, 5.1.1, 6.1.1, 8.1, 6.5.1, 6.5.2, 6.5.3; VDLUFA 2012) [21].

The rumen liquid was centrifuged, and 10 ml was used to analyse the fermentation products. The ammonia and lactate content was determined by photometric measurement at 340 nm. For ammonia, the test kit of Randox (Randox Laboratories Ltd., Crumlin, United Kingdom, Manual AM 1015) was used. The test kit of Boehringer Mannheim/R-Biopharm AG (Darmstadt, Germany) was used for lactate.

The volatile fatty acids (VFAs: acetic (AA), propionic (PA), butyric (BA), valeric (VA), isobutyric (IBA) and isovaleric acid (IVA)) were analysed using a gas chromatograph (Perkin Elmer, Clarus 580, Waltham, Massachusetts). As an internal standard, 100 μl of 2-methyl valeric acid was diluted with 250 ml of 2% metaphosphoric acid for calibration.

### DNA Extraction and Illumina Amplicon Sequencing

According to Burbach et al. [22], DNA extraction was performed using the FastDNA^TM^ SPIN Kit for Soil (MP Biomedical, Solon, OH, USA) and 200–250 mg of homogenised rumen content as starting material. The quality and purity of the DNA extracts were checked using the NanoDrop spectrophotometer (Thermo Fisher Scientific, Waltham, MA, USA).

Amplicon library preparation was performed according to Kaewtapee et al. [23] and targeting the V1-2 region of the 16S rRNA gene. Each PCR reaction mixture (20 μl) consisted of 4 μl 5x Prime Star buffer (TaKaRa Bio Inc., Kusatsu, Japan), 1.6 μl deoxynucleoside triphosphate mixture, each 0.5 μl primer (1:10 diluted), 0.2 μl PrimeSTAR HS DNA Polymerase (250U, TaKaRa Bio Inc., Kusatsu, Japan), 1 μl enhancer (BioStab PCR optimiser (II) 53833-5ML-F, Sigma-Aldrich®, Merck KGaA, Darmstadt, Germany) (only used for PCR 1), and 1 μl template DNA.

The first PCR started with an initial temperature of 95°C for 3 min, followed by 15 cycles of denaturation at 98°C for 10 s, annealing at 55°C for 10 s, extension at 72°C for 45 s, and a final extension at 72°C for 2 min. Next, 1μl of the first PCR was used for the reaction mixture of the second PCR with 20 cycles, following the same PCR conditions. The reaction mixture also contained 10 μl 5× PrimeStar Buffer, 4 μl dNTP mixture, each 1.25 μl primer (1:10), and 0.5 μl polymerase (50μl in total). Due to the nature of the samples, some samples required a pre-PCR step (10 cycles) to ensure the correct amplification.

The expected amplicons were confirmed using gel electrophoresis, normalised using SequalPrep™ Normalization Kit (Applied Biosystems) and purified using MinElute PCR Purification Kit (Qiagen), and sequenced using 250 bp paired-end sequencing chemistry on Illumina NovaSeq 6000.

Raw sequences were demultiplexed with Sabre1 and processed by Qiime2 (v.2023.5) [24]. The q2-cutadapt plugin was used to remove primers [22]. Reads were quality filtered, error corrected, dereplicated, and merged by the q2-dada2 plugin [25]. Taxonomy assignment of generated amplicon sequence variants (ASVs) was implemented in VSEARCH-based consensus and pre-fitted sklearn-based classifiers against the Silva SSU-rRNA database (v.138.1, 16S 99%) [26]. Unassigned sequences and the reads from organelles were removed. The q2-phylogeny plugin was utilised to construct a phylogenetic tree, employing MAFFT 7.3 [27] and FastTree 2.1 [28]. The phylogenetic tree, feature table, and taxonomy table were output for further statistics analysis. After filtering the data, 17,539 ASVs and 309 samples remain for further calculation.

### Statistics and Data Analysis

R version 4.3.0 was used for statistical analysis and visualisation [29]. For diversity assessment, ASV tables were rarefied to 10,000 sampling depths. Alpha diversity was estimated by Shannon’s entropy indices, and Bray–Curtis distances were calculated for beta diversity using the phyloseq R package [30]. A principal-coordinate analysis (PCoA) was utilised to ordinate the beta-diversity distances. Alpha diversity results were tested with the Wilcoxon rank-sum test, while the PERMANOVA test was used for beta diversity with 999 permutations by using Vegan R package [31]. The adjusted *p*-values were corrected for multiple comparisons using the Benjamini–Hochberg procedure. Taxonomy plot and statistics results were visualised by ggplot2 [32]. UpSet R package was used to find taxa similarity across all samples [33]. The core microbiota was identified if a genus was detected with a relative abundance of at least 1 % and 70% occurrence across all samples.

The MaAsLin2 was used to determine the association between core taxa relative abundance and crude nutrients and fermentation products. The measured values of crude protein, NFC, NDF, ammonia, AA, PA, BA, VA, IBA, and IVA were included as fixed effects [34]. SPSS (IBM SPSS Statistics Version 27.0.1.0) was used to compare the abundance means of the crude nutrients, fermentation products and microbial genera between the habitats and seasons statistically. The normal distributions were determined by the Shapiro-Wilk test. The Kruskal-Wallis analysis with post hoc Bonferroni was used for pairwise comparison of the abundance means.

## Results

### Crude Nutrients

**Fig. 1:**
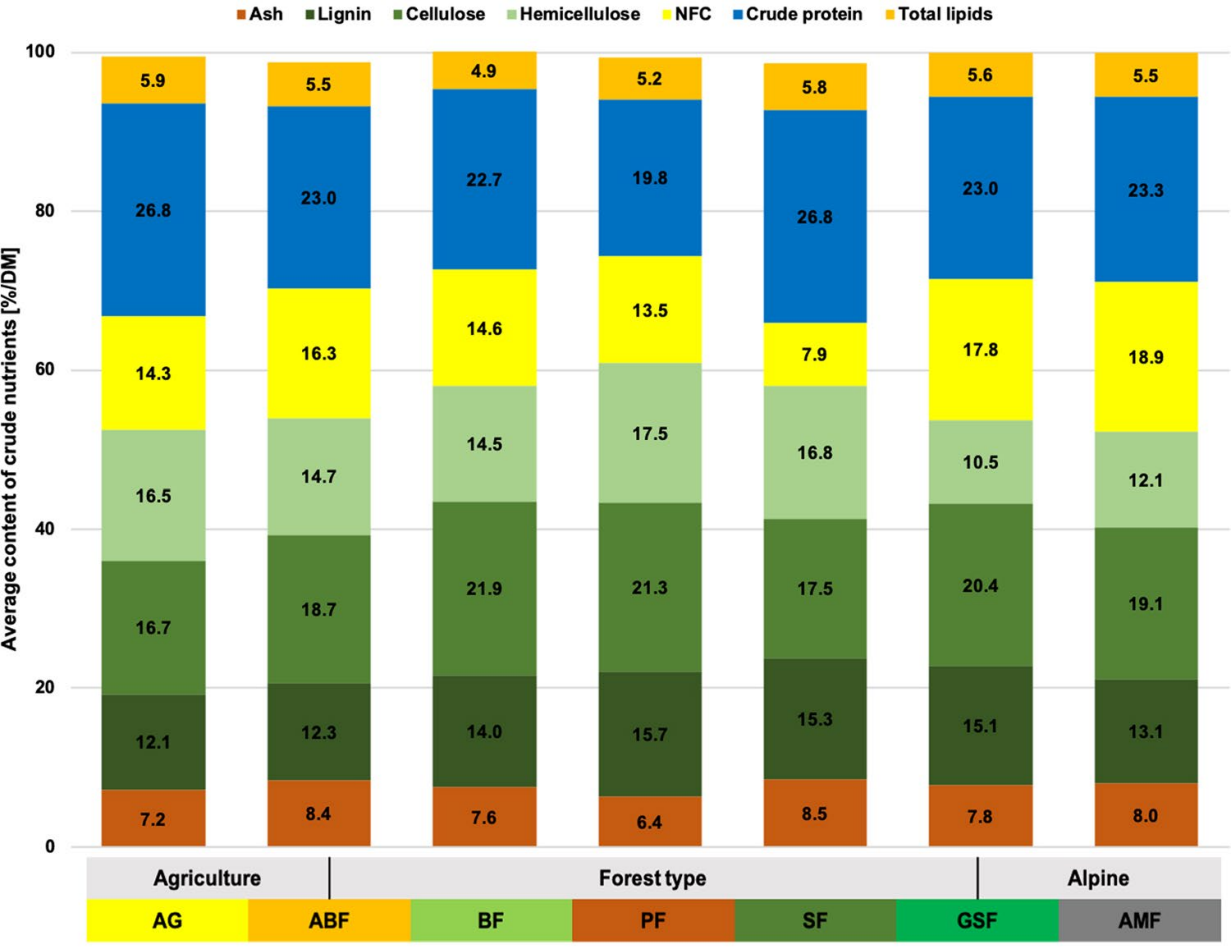
Average crude nutrient contents [%/DM] in roe deer rumen content from different habitats. Yellowish shades in the habitat legend represent agricultural habitats, greenish-brownish different forest communities and grey alpine habitats. (for further details, see Table 1). Nutrient levels were determined using Weender and VanSoest analysis. The crude protein content is composed of plant and microbial protein. NFC stands for non-fibre-carbohydrates, which mainly includes sugars and starches and pectins

All nutrient groups show significant differences in relation to the habitat factor (*p* < 0.001, Total lipids *p* = 0.037). However, the significant difference is only caused by a few habitats per nutrient group. An example of this is a significantly high crude protein content in the AG and SF habitats, a significantly high NFC content in the alpine habitats, or a significantly high total fibre content in the PF, BF, and SF forest habitats (Fig. 1, Table S4). The situation is similar for the seasonal factor (*p* ≤ 0.005), except for ash and hemicellulose. Regarding the season, the nutrient group matters greatly, but winter often determines significant differences (Table S5–S11). Gender only causes a significant difference in the ash content (*p* = 0.002). And the age class causes a significant difference in all nutrient groups (*p* ≤ 0.021) except ash and NFC. In most cases, the adult and juvenile animals do not differ significantly. Significant differences were mainly found between subadults and the two other age classes.

### Fermentation Products

**Fig. 2:**
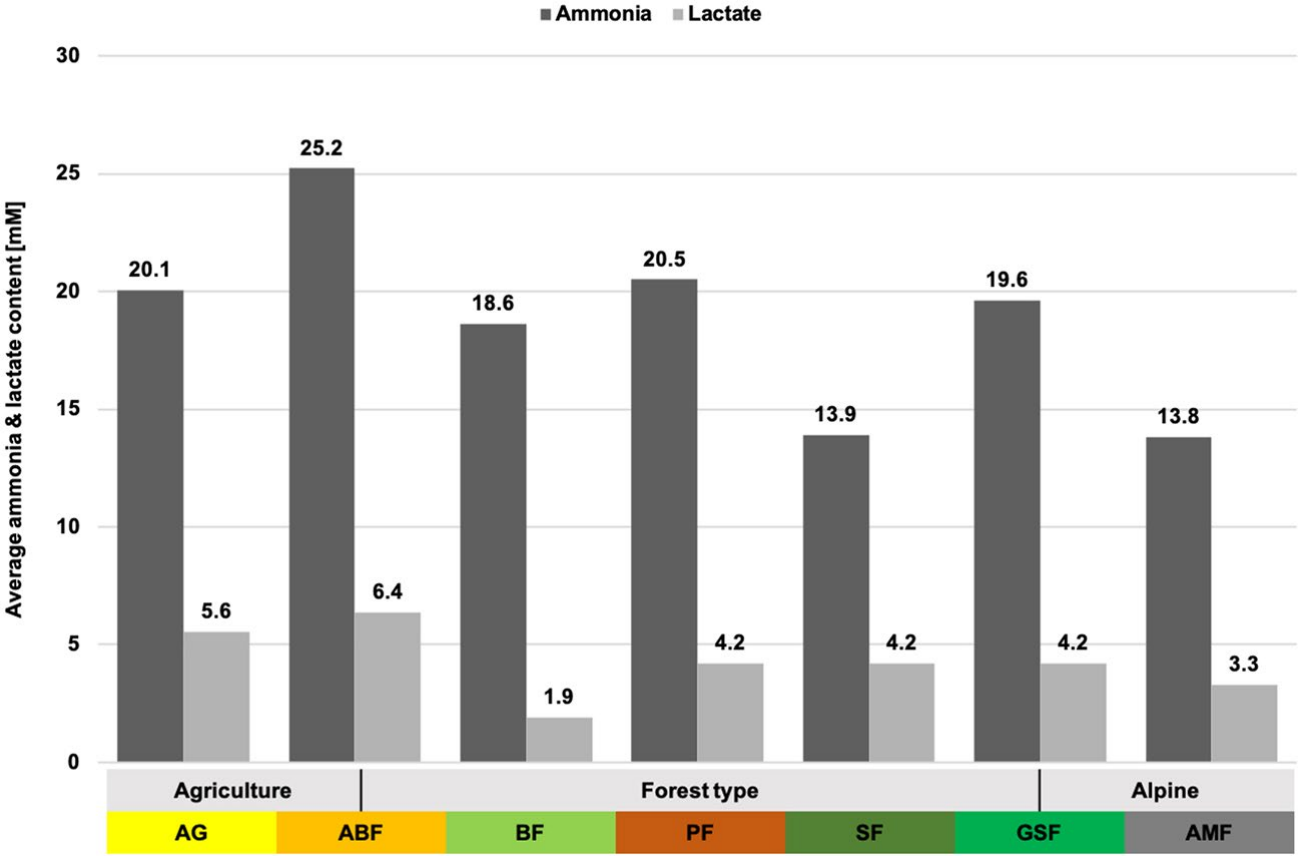
Average ammonia and lactate contents [mM] in roe deer rumen liquid from different habitats, determined by photometric measurement

Ammonia (*p* < 0.001) and lactate (*p* < 0.001) levels differ significantly between habitats (Fig. 2, Table S4). The significant differences are mainly due to the low ammonia content in habitats SF and AMF and the high content in habitats ABF and AG. The lactate content is significantly lower, especially in habitats BF and AMF and highest in habitat ABF. Gender and age class do not show any significant difference. However, the ammonia content differs significantly between the seasons (*p* < 0.001), and the lactate content does not (Table S5–S11).

**Fig. 3:**
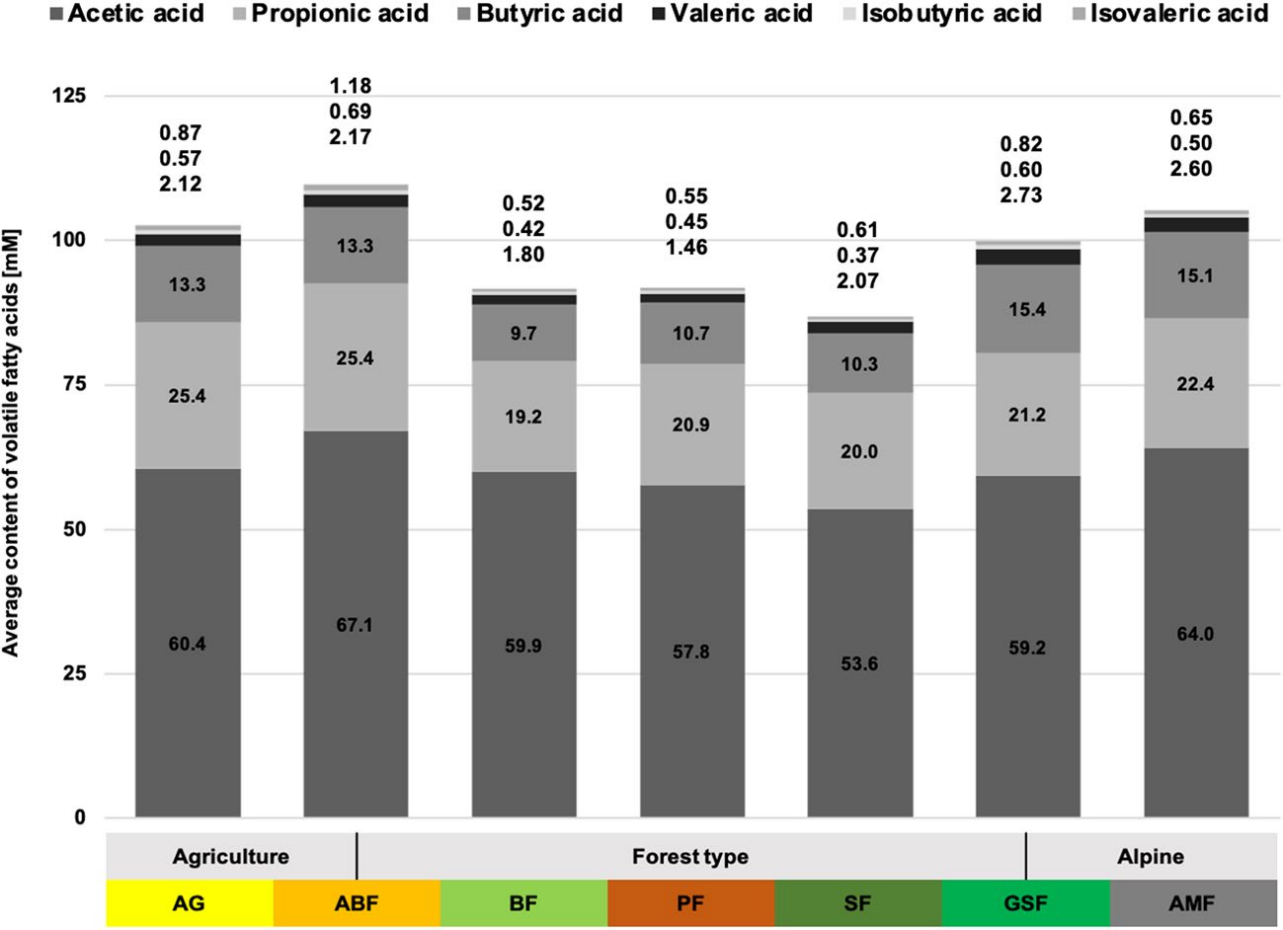
Average volatile fatty acid contents [mM] in roe deer rumen liquid from different habitats, determined by gas chromatography

There is a significant difference regarding the factor habitat for all VFAs (*p* < 0.01). This is mainly due to low levels of acetic acid in habitat SF, propionic acid in habitat BF, and butyric, isobutyric, valeric, and isovaleric acid in forest habitats BF, PF, and SF (Fig. 3, Table S4). Significantly high concentrations of acetic acid are found in ABF and AMF habitats, propionic acid in ABF and AG, and butyric and valeric acid mainly in the alpine habitats AMF and GSF. Isobutyric and isovaleric acids are highest in ABF, AG, and GSF habitats. There is also a significant difference between the age classes (*p* ≤ 0.03), except propionic acid, and the seasons (*p* < 0.001) (Table S5–S11). The significant differences between the seasons are mainly due to low values in winter for all VFAs. Gender, in turn, does not cause a significant difference in the content of VFAs.

### Habitat Portraits

#### Habitat AG (Table S4, S5)

The crude nutrients in the rumen content had a low total fibre content. The available, total fibre content was mainly characterised by a high amount of hemicellulose (16.5%), whereas cellulose with 17% and lignin with 12% are below the average. The NFC content was medium at 14.3%, and the crude protein content was the highest of all habitats (26.8%, closely followed by habitat SF). Significant seasonal fluctuations occurred for lignin and NFC. The lignin content was highest in spring (13.8%) and winter (13.8%) and lowest in autumn (9.9%). Antagonistically, the NFC content was the highest in autumn and the lowest in spring. The protein content was highest in spring at 31% and lowest in winter at 23.7%. Ammonia and lactate concentrations have also their lowest point in winter. Ammonia increased again significantly in spring, up to 24.5 mM, while lactate was still low and reached its maximum values in summer and autumn (around 7.7 mM). Propionic, butyric, and valeric acids showed similar patterns throughout the year, with the highest concentration in autumn and the lowest in spring.

#### Habitat ABF (Table S4, S6)

Overall, there was a very low total fibre content in the rumen content (46% NDF), with a peak in summer (contrary to the other habitats) and with low amounts of lignin (12.3%). Crude protein (23%) and NFC (16.3%) content were within the Bavarian average. Significant seasonal variations occurred only for the NFC content with the highest amounts in autumn (18.1%), when more tree fruits, such as acorns and beechnuts, were consumed. The average lactate concentration (6.35 mM) and propionic acid (25.4 mM) were above the average of all investigated habitats. Ammonia concentration was the highest of all habitats, with 25.2 mM. Iso-butyric acid (0.69 mM) and iso-valeric acid (1.18 mM) were also significantly enhanced compared to other habitats.

#### Habitat BF (Table S4, S7)

The total fibre content in the rumen was relatively high with 50.2%; NFC (14.6%) and crude protein (22.7%) were in the medium range compared to other habitats. We found significant seasonal differences in fibres (especially hemicellulose), proteins, and NFCs. Crude protein and NFC increased significantly during this season. Lactate (1.92 mM), propionic (19.2 mM), butyric (9.7 mM), and isovaleric acid (0.5 mM) occur in very low concentrations.

#### Habitat PF (Table S4, S8)

The rumen content had significantly high total fibre contents (54.5% NDF), and all fibre fractions had their highest proportions in this habitat. The NFC content is only 13.5%, and the crude protein content was significantly low and the lowest of all habitats, with 19.8% on average. The proportion of valeric acid was also very low, with only 1.46 mM. Strong seasonal fluctuations occurred, especially in the fibre content (cellulose and lignin) and crude protein, as well as in some fermentation products. NDF, cellulose, and lignin reached their maximum value in winter and their lowest value in summer. The course of the crude protein content is antagonistic to this. Hemicellulose content was highest in spring.

#### Habitat SF (Table S4, S9)

The total fibre content in the rumen was relatively high, with 51% NDF, with comparatively few celluloses and more hemicellulose. The crude protein content was nearly the highest with 26.78%, just behind habitat AG, and the NFC content was the lowest of all habitats with 7.9% (peak in autumn with 9.4%). The course of crude protein and total fibre content were antagonistic, with the highest content of total fibre in winter and the lowest in summer. The proportion of cellulose (17.5%) was comparatively low compared to other habitats, and the proportion of hemicellulose (16.8%) was high. The curves of the fibre fractions were similar throughout the year, except for summer. While hemicellulose and lignin reached their lowest value here, the cellulose fraction was high in this season. In addition, the concentration of ammonia was the lowest at 13.9 mM. Significant seasonal differences were only found for total lipids and some fermentation products.

#### Habitat GSF (Table S4, S10)

The total fibre (46% NDF) and crude protein content (23%) was slightly below average (∅ 23.6 and 48%). On the other hand, the NFC content was high, with nearly 18%. Regarding the fibre fractions, the hemicellulose content was the lowest of all habitats (10.5%). In contrast, the lignin (15.1%) and the cellulose content (20.4%) were quite high. The total fibre content (NDF), as well as all fibre fractions, had their peak values in winter. Cellulose had a second peak in summer. The crude protein content was highest in spring. Moreover, the butyric acid (15.4 mM) and the valeric acid content (2.7 mM) were the highest of all habitats. Seasonal variations occurred mainly in protein and total fibre content, as well as in some fatty acids.

#### Habitat AMF (Table S4, S11)

The proportion of total fibres was low (44.3% NDF); NDF and all fibre fractions had the highest content in winter. The crude protein content was medium (23.3%), with a peak in spring, and the NFC content was highest in this habitat (18.6%), with the highest proportions in summer and autumn. Furthermore, the concentration of butyric acid (15.1 mM) and valeric acid (2.6 mM) was high, and the concentration of ammonia (13.8 mM) and lactate (3.3 mM) was relatively low. The typical strong seasonal fluctuations in the alpine region were also reflected in the distribution of nutrients (Fig. 5). Except for NFC and hemicellulose, all groups have significant seasonal differences.

### Variation in the Composition of the Rumen Microbiota

**Fig. 4:**
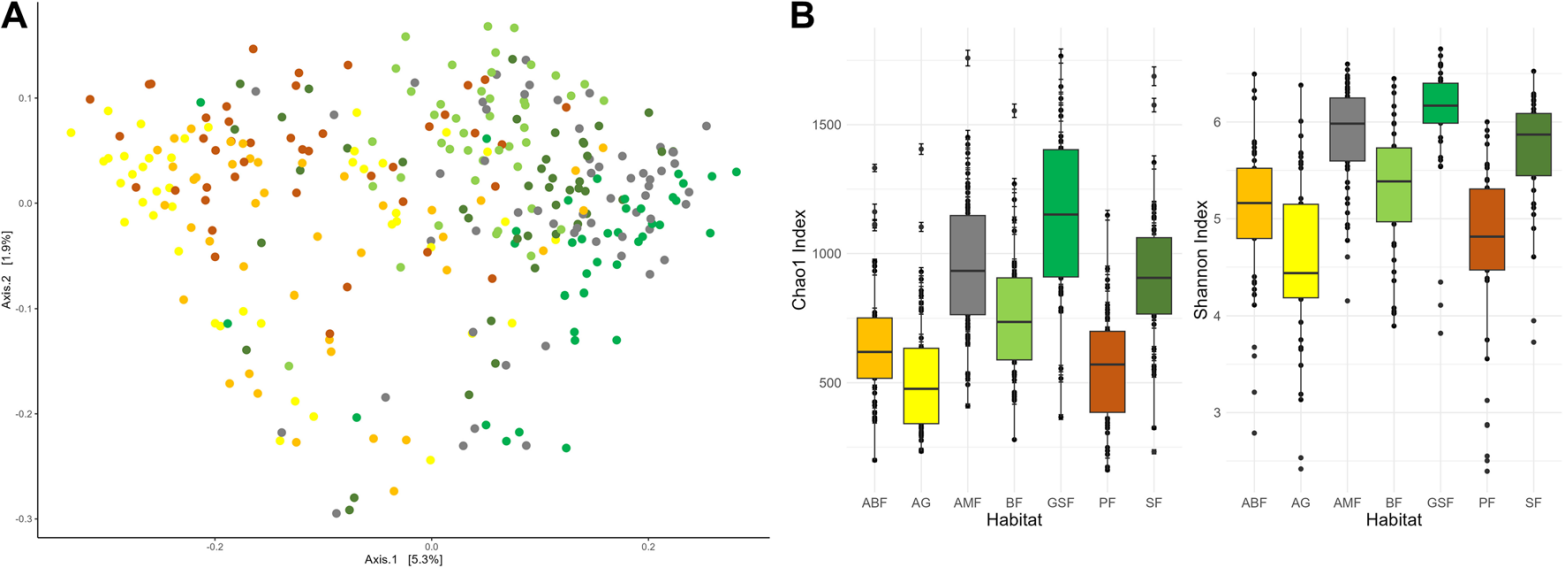
**A** Principal coordinate analysis (Bray Curtis distances) with the distribution of rumen microbiota samples per habitat (Permanova test: *p* = 0.001) **B**: Chao1 and Shannon diversity index per habitat (*p* < 0.001)

The bacterial microbiota in the rumen content of roe deer differed significantly in terms of habitat (*p* < 0.01), season (*p* < 0.01), and age class (*p* < 0.01, adult vs subadult vs juvenile). Most significant differences exist between the juvenile and subadult age classes. Gender was not causing a significant difference. The principal coordinate analysis (PCO) (Fig. 4A) showed the clustering of samples from habitats SF, GSF, AMF, and BF on the right side of the plot. The smallest distance is between habitat GSF and AMF. The second cluster of similarity is formed among habitat AG, ABF, and PF on the left side of the plot. Alpha-diversity analyses (Fig. 4B) showed that habitat GSF has the highest ruminal microbial Chao1 and Shannon index and habitat AG has the lowest. A Wilcoxon rank-sum test revealed significant differences between several pairwise results (see Table S1).

**Fig. 5:**
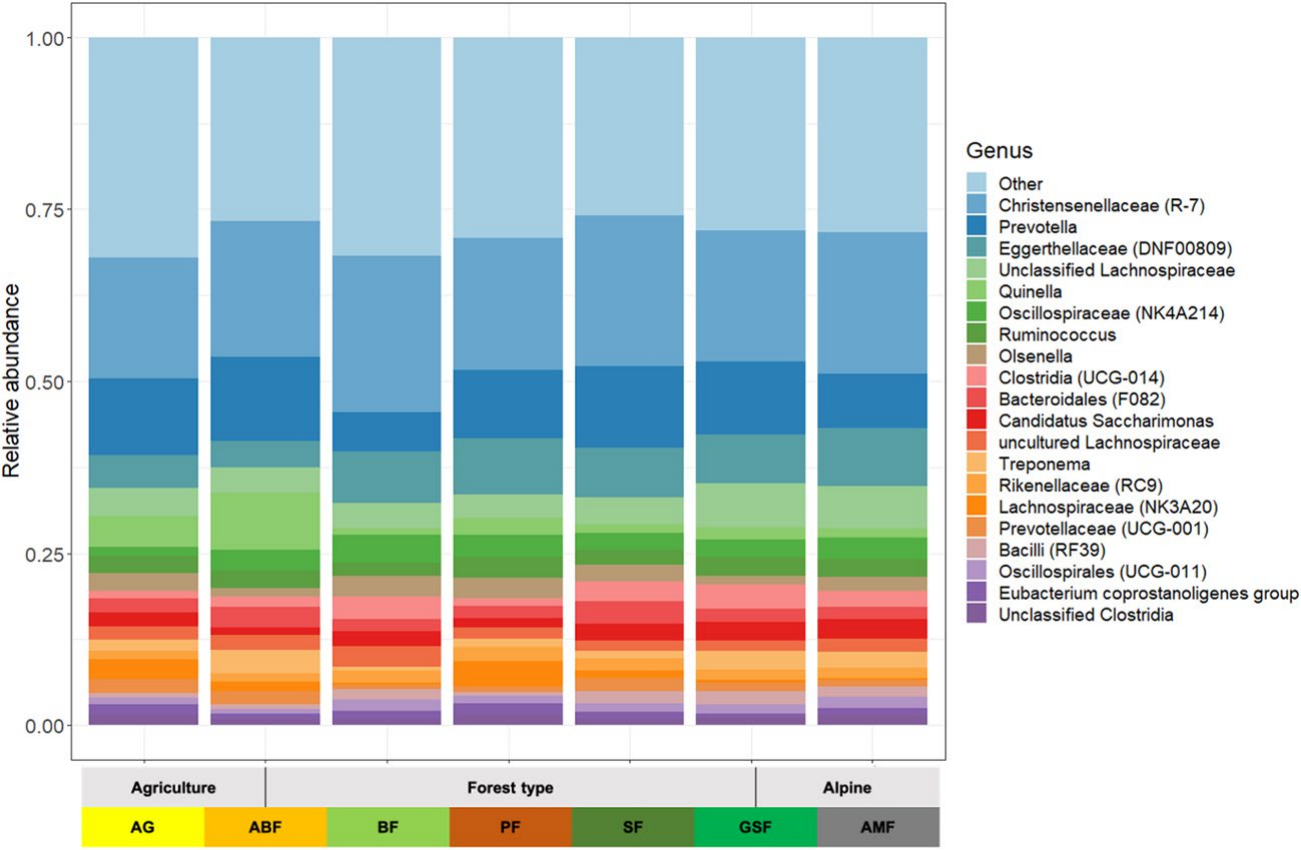
Distribution of the top 20 microbiota representatives per habitat

**Fig. 6:**
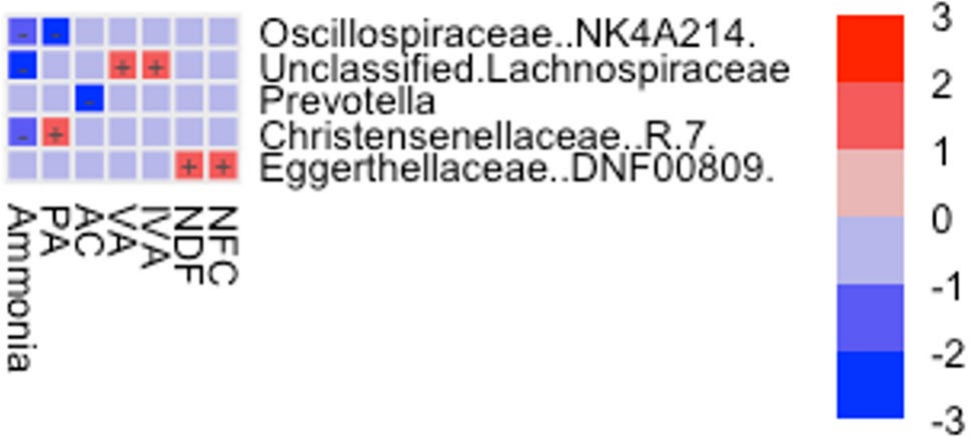
Heatmap of the significant associations (-log(qval)*sign(coeff)) between the five members of the core microbiota and the crude nutrients and fermentation products. Positive or negative signs indicate an enrichment or decrease in the corresponding genera in parallel with increasing nutrient contents and fermentation products. The analysis was performed with the MaAsLin2 R package

On average, *Firmicutes* (*Bacillota*) is the most frequent phylum in the roe deer rumen content (61%), followed by *Bacteroidota* (19%) and *Actinobacteriota* (14%) (Table S12).

Two hundred seventy-seven genera could be identified, from which 163 genera were detected in the rumen contents of all habitats (Fig. 5, Fig. S13). The remaining 114 genera were only found in some habitats and a few only in one of them (Fig. S2). Fifty-four of 277 genera could not be classified at the genus level but at higher taxonomic levels, showing the high number of still unknown genera in the roe deer rumen content.

Overall, the *Christensenellaceae* *(R-7)* is the most common genus with an average relative abundance of 20%, and it, therefore, is also the leading contributor to the, so-called core microbiota [35]. The common core microbiota of our roe deer consists of 5 genera, forming 44% of the total microbiota (Table S13). The identified genera are *Eggerthellaceae (DNF00809)*, *Prevotella*, *Christensenellaceae (R-7)*, *Unclassified Lachnospiraceae*, and *Oscillospiraceae (NK4A214).*

Significant differences between habitats can be found for most genera (Table S14). *Christensenellaceae (R-7)*, as some representative of the core microbiota, shows no significant difference between the habitats. The significant difference for the genus *Prevotella* is due to the low values in the beech forest habitat (BF), whereas *Oscillospiraceae (NK4A214)* is more common there. In the agricultural habitat (AG), on the other hand, they showed a significantly low abundance. *Eggerthellaceae (DNF00809)* occur significantly less in agricultural-dominated areas and have their highest values in the pine (PF) and alpine mountain forest habitat (AMF). In the two alpine habitats (GSF and AMF), the uncl. *Lachnospiraceae* occur in significantly high abundances.

In addition to the core microbiota, some other genera, such as *Fretibacterium*, *Latilactobacillus*, *Syntrophococcus*, *Streptococcus*, *Lentilactobacillus*, *Ralstonia*, *Tyzzerella*, *Catenisphaera*, *Enterococcus*, and *Leuconostoc,* only occur in 1–2 habitats with increased abundance. Alternatively, *Quinella* or *Treponema* show a strikingly low abundance in one habitat (BF).

A MaAsLin2 analysis showed significant associations between the top representatives of the microbiota and some crude nutrients (CP, NDF, NFC) and fermentation products (Fig. 6).

## Discussion

The present study investigated 311 rumen contents and the microbiota of wild roe deer living in seven different habitats across different seasons, animal ages, and gender. Especially based on a large number of samples, this study provides many more insights about wild ruminants and their microbiome as the number of studies is limited in contrast to domesticated ruminants. To the best of the author’s knowledge, only four publications studied European roe deer using amplicon sequencing methods [19, 36–38], and one the Chinese roe deer [39]. These studies mainly lack a representative number of rumen samples varying between 3 and 19. Studies with larger numbers of samples from deer were only carried out with faecal samples (red deer, 136 faecal samples) [40].

The challenge with relatively unexplored microbiomes is a large number of unclassified species. Previous studies in wild animals found a considerable number of unclassified OTUs [19, 35, 41, 42], as observed in the present study. Similarities were found at the phylum level between the present and previous studies focused on the roe deer ruminal microbiome by Ricci et al. [19] and Wilson et al. [37] and also in the rumen content of other wild ruminants, such as reindeer, elk, bison, moose, red deer, and sika deer [40, 42–48]. *Firmicutes* (*Bacillota*) was the dominant phylum, followed by *Bacteroidota*.

The rumen microbial ecosystem is dominated by a core community comprising *Prevotella*, unclassified *Clostridiales*, unclassified *Bacteroidales*, unclassified *Ruminococcaceae*, unclassified *Lachnospiraceae*, *Ruminococcus*, and *Butyrivibrio*, known as the “core microbiome”, which is found in domesticated and wild ruminants around the world [35, 41]. All these genera are present in the samples, but an exact comparison of the genera is not possible due to a deeper taxonomy classification. The core microbiota of the current Bavarian roe deer study contains five genera. The most frequently represented genus in the present samples is *Christensenellaceae* (R-7). In the worldwide comparison, *Prevotella* is in the lead, which is the second most common species in the roe deer samples.

The observed differences in microbial composition due to various habitats and the apparent impact of the forage were also studied for other wild ruminants, including reindeer and moose [42, 43], but mostly selectively composed diets were used [19, 49]. A high proportion of concentrate feed often characterises these. In other publications, natural forage was compared with selectively composed diets in the husbandry of actual wild ruminants [19, 40, 45, 47, 48, 50, 51].

The significant difference between the age classes can be explained by the animals eating different forages at different stages of development. It should be noted, however, that the data on juveniles are only available from September to April due to hunting seasons. Seasonal effects are caused by the sometimes strongly differing forage availability per season and were already analysed for Sika and White-tailed deer [52, 53].

In this study, we compared roe deer from seven different habitat types, representing Bavaria’s most characteristic and extreme habitats. The analysis of the crude nutrient and the plant species composition in the rumen contents showed that the roe deer are confronted with very different forage offers. This is also reflected in the ruminal microbiota. The average cross-habitat crude protein content is 23.6%. This is slightly lower than in the previous study (27.4%), in which only two habitats were investigated [3]. It must be considered that a large part of this is microbial protein and that vegetable protein is quickly converted into ammonia. A confirmation of that is that the ingested plants have significantly lower protein contents (coniferous trees: ~12.9%/DM; shrubs: ~17.3%/DM; herbs: ~19.4%/DM; grasses: ~3–12.6%/DM [54, 55]). Furthermore, nitrogen can be recovered via the ruminal-hepatic cycle. The average NFC content of 14.9% was higher than in the previous study (11.1%). As expected, the average fibre content (47.9% NDF, 27.8% CF) was high and corresponded to the values from the preliminary study (47.6% NDF, 27.8% CF). The protein content in the rumen content always reaches its minimum, and the total fibre content is at its maximum in winter (with the only exception: habitat ABF), regardless of how large the respective proportions are in the habitat as a whole. However, there are big differences between the individual fibre fractions.

Thanks to recent findings and the switch from the rigid classification into feeding categories, it is known that roe deer are pretty tolerant of high fibre content. This does not only apply to so-called intermediate types or roughage eaters [3, 14]. However, there are many differences between the seven habitats, some of which are significant, and each habitat has its own specific browsing range and nutrient profile. Commonalities, as demonstrated in Fig. 4, were found among all forest habitats (beech, spruce, grassland spruce and mountain forest), which are dominated by similar tree species, as well as shrub and dwarf shrub communities. Furthermore, the agricultural and agricultural beech habitats show strong similarities in cultivating the agricultural areas and the field edge vegetation. The pine habitat forms more of a cluster with the two agricultural habitats, which may indicate a tendency toward monoculture. The pine stands here are almost pure, and the ground vegetation is relatively poor in species. Likewise, the plant culture in the agricultural area is very one-sided in larger sections. Further correlations can be seen in the Shannon diversity index (Fig. 4 B), which is higher in the alpine habitats GSF and AMF, which could indicate adaptation to higher altitude habitats. Inhabitants of high altitudes have been described as having a more diverse microbiome than inhabitants of lowlands [56].

Interrelationships can also be depicted at the functional level. Functional relationships between microbial abundance in the rumen content and nutrient supply can be identified in several habitats. A correlation between nutrients, fermentation products, and the core microbiota members was shown for individual genera. Still, not all of them can be explained based on the literature. The genus *Prevotella*, for example, shows a significant decrease in association with increasing acetic acid, which is fibre-associated, whereas *Prevotella* is clearly protein-associated. The significant decrease in *Oscillospiraceae (NK4A214)* associated with increasing propionic acid results from cellulolytic properties [57], mainly increasing acetic acid production. *Christensenellaceae (R-7)* is also described in the literature as an acetic acid producer and fibre utiliser [57], so the positive correlation with propionic acid cannot be explained or must be influenced by unknown factors. The ammonia found in rumen contents is primarily the product of the breakdown of vegetable protein. A negative correlation with mainly fibre-associated species, like *Christensenellaceae (R-7)*, *Oscillospiraceae (NK4A214)*, and *unclassified Lachnospiraceae* [58], can therefore be explained.

The *Eggerthellaceae (DNF00809)*, the core microbiota’s last representative, are significantly increasing with increasing NFC and NDF content. The *Eggerthellaceae* are known for degrading polyphenols [59], which could explain the association with fibre content. Another known function of the *Eggerthellaceae* is their involvement in maintaining homeostasis [60], which could explain a link with NFC content.

Across all habitats, a very differentiated, habitat-specific composition of the microbiota can be identified. Even four of the five taxa of the core microbiota show significant differences between the habitats. Not all habitat-specific distributions can be explained, as there are still many unclassified genera in the microbiome of wild ruminants whose functional assignment is unclear. However, some clear correlations can be shown between the available forage, the nutrients thus consumed, and the resulting microbiota.

The agriculture habitat (AG) was characterised by high protein availability, and hemicellulose dominated the fibre fractions. Hemicellulose is found in large amounts in field crops and sweet grasses, an essential part of the forage in this habitat. The lignin content was highest in winter and spring when many woody branches and shrubs were eaten. In autumn, on the other hand, the NFC content dominates due to tree fruits, which are increasingly found in the tree communities at the edges of the fields [6]. Accordingly, proteolytic genera such as *Prevotella, Prevotellaceae (UCG001)* [61], and *Family XIII AD 3011group* [62] play an important role here. *Prevotella* also has hemicellulolytic properties [61]. In contrast, *Ruminococcus*, *Oscillospiraceae*, and *Clostridia* are specialised in fibre utilisation [63, 64], especially cellulose [58], were present in significantly low numbers. *Tyzzerella* is described as a pathogenic bacterium [65] and occurs more frequently in spring. From winter to spring, the microbiota in the habitat AG changes very strongly (see Fig. S3). The increased occurrence of Tyzzerella at this time could possibly be connected to a microbial imbalance.

The Agriculture-Beech-Forest habitat (ABF) included large areas of agricultural land but also beech and oak-hornbeam forests [66]. The total fibre content in the rumen contents was relatively low. Protein and NFC content were in the mid-range. The NFC content was highest in autumn when the deer could browse the numerous beech and oak tree fruits [66]. These were available in large quantities, especially in the sample year 2018, as this was a fattening year for both species. The particularly high levels of ammonia, lactate, and some VFAs were also striking. This could explain the importance of the *Ralstoni*a genus. *Ralstonia* is known to utilise VFAs and is capable of denitrification under anaerobic conditions [67, 68]. An N surplus in the course of the harvests could be an explanation for particularly high *Ralstonia* proportions in autumn. The proteolytic and pectinolytic genera *Prevotella*, *Prevotellaceae (UCG 001)*, and *Prevotellaceae (UCG 003)* are also found in significant abundance in this agricultural habitat. As an important cellulolytic genus, *Treponema* [69] occurs in significantly high abundances in this habitat.

Large areas of pure beech forest are also located north of Bavaria, as in habitat beech forest (BF) [70, 71]. The rumen content contained significantly more total fibres with a high cellulose content. The protein content was in the medium range, and the NFC content was slightly below average. The seasonal fluctuations in this habitat were strongly reflected in the crude nutrient profile. A large proportion of shrubs (mainly) in the rumen contents dominated almost at all times of the year; only in spring do the conifers predominate, when presumably only a few deciduous shrubs are still to be found. Accordingly, high total fibre contents are found in the rumen content in winter and spring. Appropriately, the proportion of *uncultured Lachnospiraceae* and *Enterococcus*, which have fibrolytic properties [58, 72], occurs in significantly high abundances*.* In summer, tree and shrub fruits and herbs are added, which explains the crude protein and NFC peak in summer. The NFC content remains relatively high even in autumn when abundant beechnuts are available to the animals. The low proportion of *Prevotella, Prevotellaceae (UCG 001)*, and *Prevotellaceae (UCG 003)* can be explained by the overall relatively low crude protein content and higher *Olsenella* (glucose fermenters) and *Latilactobacillus* (saccharolytic) proportions [73, 74], possibly by the higher sugar content in the forage in the summer months.

Another extreme habitat is the almost pure pine forests (PF) in the Upper Palatinate Basin [75, 76]. Accordingly, strong seasonal fluctuations in the nutrient profile are also reflected here. In this habitat, the deer mainly use dwarf shrubs, especially blueberry. Tree and field fruits, berries, cherries and apples are also on the otherwise very fibrous menu, depending on the season. The crude nutrient profile shows significantly high total fibre values, which, as expected, are highest in winter and lowest in summer. Again, the crude protein content is highest in summer, when shrubs, trees, and field crops supplement the forage. A clear connection can be drawn between the high proportions of *Ruminococcus*, which show their highest values in winter, parallel to the fibre values. The increased starch content of crops such as maize can explain the high proportion of *Streptococci* in summer. For the *Eggerthellaceae (DNF00809)*, it is known that an essential function of genera belonging to this family is the maintenance or support of homeostasis [60], and they are increased by stress exposure. Since this genus is also more abundant in summer, this could be related to the forage’s significantly higher protein and starch content in summer. Genus *Syntrophococcus* is another fascinating bacteria with the highest abundance in habitat PF (1.15%). These bacteria can demethylate lignin [77]. The breakdown of lignin is otherwise only known from anaerobic fungi but not from rumen bacteria.

In contrast, the spruce forest habitat SF in the south of Munich [78] has high crude protein content in the nutrient profile. The total fibre content is also high over the year, with a high proportion of hemicellulose and lower cellulose content. Especially in winter, the animals must resort to coniferous wood, while herbs strongly dominate the forage composition the rest of the year. This explains the high protein content, which peaks in summer. In addition to deciduous wood and shrubs, the forage is strongly supplemented by forbs, cryptogams in autumn, and field crops in summer. The high protein content probably also explains the high proportion of proteolytic *Prevotella*, *Prevotellaceae (UCG 001)*, *Prevotellaceae (UCG 003)*, *Butyrivibrio*, and *Eubacterium nodatum group* [61, 79–81]. Most of them can also utilise hemicelluloses well [61, 80].

Characteristic Bavarian habitats are the alpine areas, which roe deer use up to a certain altitude. An important pre-alpine area is grassland farming, which is complemented by forest areas [82]. The Grassland-Spruce-Forest habitat (GSF) has a high NFC content in the nutrient profile. The total fibre and crude protein content are average. The total fibre content is highest in winter and lowest in spring. The lignin content is comparatively high, as is the cellulose content. In contrast, the crude protein content is highest in spring. The high protein content in spring can probably also be explained by the increased browsing of herbs in this habitat. However, the correlation between the botanical rumen content analysis (BRCA) and the crude nutrient profile must be considered with reservations, as only 13 samples from the BRCA were examined in this habitat. Moreover, these were mainly from the winter months, which also explains the relatively high proportion of anthropogenic bait feed in the form of apple pomace in the rumen contents. Significantly high abundances of cellulolytic *unclassified Lachnospiraceae*, *Treponema*, and *Eubacterium hallii group* can be explained by the high cellulose contents [58, 69, 79]. The saccharolytic genus *Marvinbryantia* [69] also has significantly high proportions, which fits with the high NFC values.

The Alpine Mountain Forest habitat (AMF) is in the middle of the mixed mountain forest of the Ruhpolding Limestone Alps [83]. Due to the alpine location, the animals living here must be able to adapt quickly to changing weather conditions and endure distinct seasons. The NFC content is also high in this alpine habitat; in fact, the highest proportions of all are found here. The total fibre content is relatively low, and the crude protein content is average. The total fibre content is only high in winter and low the rest of the year. The fractions are dominated by cellulose and lignin. The crude protein content is highest in spring, as in Habitat GSF. The NFC content is consistently high. Shrubs also dominate the food choice in this habitat, but the selection of shrubs consumed is much more diverse than, e.g. in habitat BF or PF. In summer and autumn, fruits also play a major role. A significantly increased proportion of *Marvinbryantia* is probably due to a starch- or sugar-associated forage. High proportions of *Eggerthellaceae (DNF00809)* and *Enterorhabdus* could be related to presumably high proportions of plant secondary metabolites in the pronounced herbaceous layer and various woody branched plants [84]. However, exact proportions have not yet been examined but will be part of the following analyses. Significantly occurring fibre-associated genera in this habitat are *unclassified Clostridia*, *unclassified Lachnospiraceae*, and *Prevotellaceae (UCG 003).*

In order to understand the needs or adaptive mechanisms of roe deer, this should be considered in the context of their surrounding habitat. The roe deer is highly and rapidly adaptable to the changing forage availability, contributing to these animals’ distribution success. However, they are also very well adapted to their habitat to make the best possible use of the given forage, even in months of privation. As we could already show in our preliminary study based on the energy density in the habitats, roe deer cope well with the given forage offer and adapt to less energy-rich food, e.g. by an increased rumen filling [4]. The energy densities from all the habitats studied are still being evaluated. Still, the animals’ weights already indicated no malnutrition or insufficient supply in any of the habitats. And results of the actual study show that the energy densities and the animals’ weights indicated no malnutrition or insufficient supply in any of the habitats [85]. For example, if we consider additional feeding in winter, habitat-specific adaptations should definitely be considered.

The dynamic changes in the microbiome structure support the colonisation of new habitats. Adaptation to a particular forage per se is, of course, also determined by their anatomy and by the environment in which they live, but we can see that the roe deer is a tolerant animal with strong dietary deviations involving plant species high in nutrients. The limits of this research were that due to the new nature of the study, several bacterial species have not yet been cultured; therefore, there were a considerable number of unclassified/unknown taxa among the sequencing data. However, this study opens the possibility of understanding the interplay between microbiome, crude nutrients, and VFAs. A future perspective will be to culture anaerobic species from the rumen of wild animals to better classify them.

### Supplementary Information

ESM 1: (PDF 1145 kb)

## Data Availability

The microbiota dataset analysed during the current study is available in the ENA repository, no. PRJEB61103.
