# Supplementary material for: Microbiota and Nutrient Portraits of European Roe Deer (Capreolus capreolus) Rumen Contents in Characteristic Southern German Habitats
Source: Microb Ecol. 2023 Oct 24;86(4):3082–96. doi: 10.1007/s00248-023-02308-5 (PMC10640537; doi:10.1007/s00248-023-02308-5)
Supplement: Supplementary file 1 — (PDF 1145 kb) [file 248_2023_2308_MOESM1_ESM.pdf]

Supplementary Information

Microbiota and nutrient portraits of European roe deer (*Capreolus capreolus*) rumen contents in characteristic southern German habitats

Sarah-Alica Dahl[1], Jana Seifert[2,3], Amélia Camarinha-Silva[2,3], Yu-Chieh Cheng[2,3], Angélica Hernández-Arriaga[2,3], Martina Hudler[4], Wilhelm Windisch[5], Andreas König[1]

[1] Technical University of Munich, Wildlife Biology and Management Unit, Chair of Animal Nutrition and Metabolism, Hans-Carl-von-Carlowitz-Platz 2, 85356 Freising, Germany
[2] University of Hohenheim, HoLMiR – Hohenheim Center for Livestock Microbiome Research, Leonore-Blosser-Reisen-Weg 3, 70599 Stuttgart, Germany

[3] University of Hohenheim, Institute of Animal Science, Emil-Wolff-Str. 10, 70599 Stuttgart, Germany

[4] Weihenstephan-Triesdorf University of Applied Sciences, Game Management and Wildlife Management, Hans-Carl-von-Carlowitz-Platz 3, 85354 Freising
[5] Technical University of Munich, TUM School of Life Sciences, Liesel-Beckmann-Straße 2, 85354 Freising

Corresponding author: Sarah-Alica Dahl, sarah.dahl@tum.de

Botanical rumen content analysis

The browsing was analysed by botanical rumen content analysis (BRCA). For this purpose, a part of the homogenized rumen content was rinsed with distilled water using wet sieving and three sieves with different mesh sizes (6.3, 3.55 and 1mm) [1, 2]. The macroscopically determinable parts were collected in sieves 1 and 2. In the third sieve, the smallest, macroscopically indeterminable solid components collected. The determinable plant parts were macroscopically determined at genus name and, if possible, at species level and assigned to defined browsing categories (see Fig. S1). They were then dried for 24 hours at 60°C and the percentage of the determinable mass was calculated.

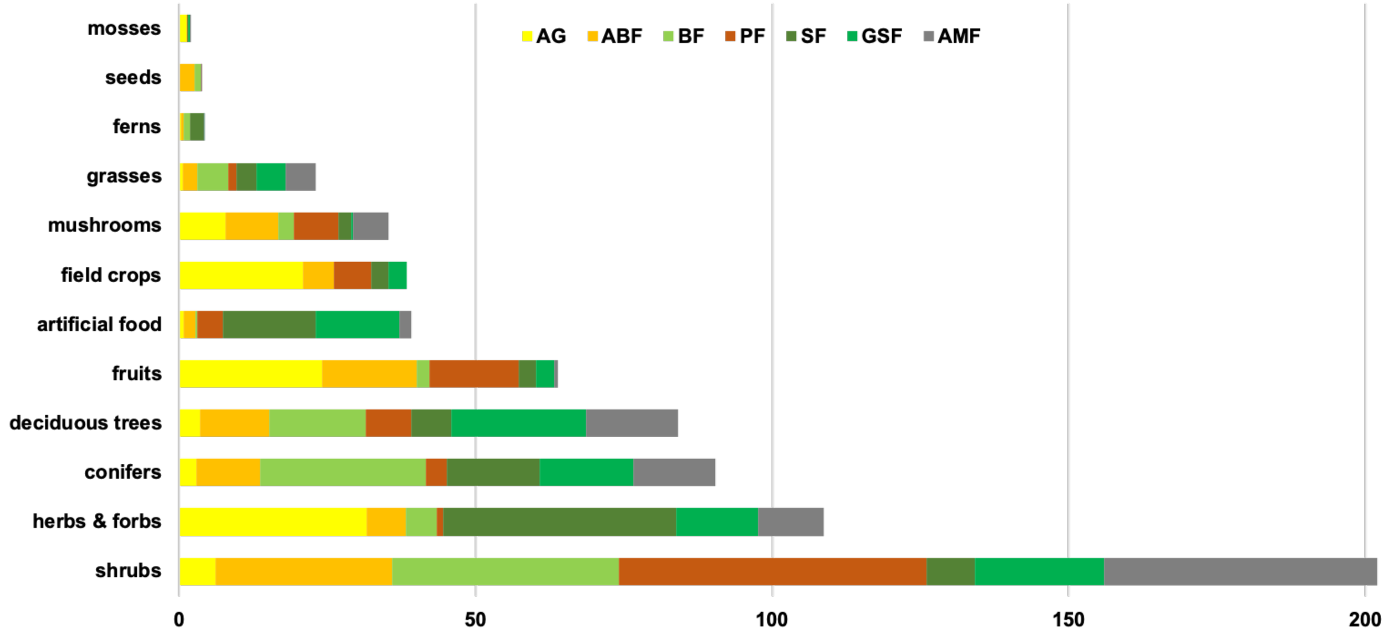

Fig. S1 Results of the botanical rumen content analysis, average content of plant groups of the determinable plant mass in the rumen content per habitat [%]

Additional explanations and information regarding the results of the botanical rumen content analysis

Agriculture habitat - Roe deer in this habitat consumed a conspicuous amount of tree and field fruits, as well as herbs, and comparatively little deciduous and coniferous wood. Sweet grasses and shrubs were only found in higher proportions in the rumen content in spring with 14% each [2].

Agriculture-Beech-Forest habitat - The ingested food was very diverse, in contrast to some other habitats, such as habitat PF. All forage categories were represented, albeit in very different proportions. In addition to the dominant shrubs, roughly equal proportions of deciduous and coniferous wood were consumed, as well as a high proportion of tree fruits. The consumed fibres consist mainly of non-woody shrubs such as blackberry, blueberry or raspberry. In addition to acorns and beechnuts, which had a mast year in 2018, cherries and apples as well as shrubbery fruits were also included. The agricultural part of this habitat also required the intake of field and arable crops, grasses and forbs.

Beech forest - The BRCA revealed a rather undiversified mixture in the rumen content, mainly consisting of shrubs, coniferous wood and deciduous wood. Another striking feature was the increased proportion of mushrooms (∅ 5.2%) in the rumen content, in contrast to other habitats like AL, PF or ABFM. Shrubs, especially blackberry, were a dominant component of the ingested forage, especially in winter and autumn. In summer, the shrub fruits were crucial, as well as mushrooms and herbs. Over the winter, shrubs were almost completely eaten away (Undocumented reports), so that in spring a lot of coniferous wood still dominated the forage until shrubs, deciduous wood and herbs sprout freshly. However, coniferous wood also played an important role in winter with 41%, and the fibre values were accordingly highest in these two seasons. Mushrooms, with the exception of spring, seemed to be a protein- and carbohydrate-rich supplement all year round.

Pine forest - The forage in the rumen content was very strongly dominated by shrubs (mainly blueberry) in all seasons and an increased proportion of fruits (shrub, tree and field fruits/crops, like berries, cherries, apples, some acorns and corn), mainly in summer with 31.2%. All other categories of forage were only consumed by the animals in very small proportions.

Spruce forest - The rumen content was dominated by conifers, herbs and shrubs, depending on the season [2]. Herbs were an essential part of the forage during their vegetation period and they dominated the rumen content with over 30 and 50% in spring and summer, respectively. In spring, large amounts of coniferous wood and shrubs were also consumed. During winter, the forage consisted mainly of conifers, followed by shrubs. During the transition in autumn, the proportions were more balanced and a mixture of forbs, herbs, cryptograms, hardwoods and shrubs were consumed.

Grassland-spruce-forest habitat - Shrubs, coniferous wood and deciduous wood dominated the rumen content. In addition to anthropogenic bait feed, herbs, field crops, mushrooms, forbs and shrubs and tree fruits were also found.

Alpin Mountain Forest - The rumen content was dominated by shrubs. Far behind are deciduous and coniferous woods, as well as herbs and mushrooms. Blackberry (*Rubus* sp.) was also eaten as a preferred shrub in this habitat, but did not dominate the category as much as in other habitats, like BF. Blueberry (*Vaccinium myrtillus*), dog rose (*Rosa canina*) or wayfarer (*Viburnum lantana*), for example, were also browsed.

Table S1: Significant pairwise results of the Wilcoxon rank-sum test of Shannon-diversity of the microbiota

	GROUP1	GROUP2	P.ADJ
1	AG	ABF	9.19E-03
2	AMF	ABF	1.60E-07
3	BF	ABF	1.45E-01
4	GSF	ABF	4.30E-09
5	PF	ABF	8.23E-02
6	SF	ABF	1.95E-05
8	AMF	AG	5.93E-11
9	BF	AG	1.60E-04
10	GSF	AG	3.23E-11
11	PF	AG	1.82E-01
12	SF	AG	2.45E-09
15	BF	AMF	1.95E-05
16	GSF	AMF	3.32E-02
17	PF	AMF	8.25E-11
18	SF	AMF	1.25E-01
22	GSF	BF	1.60E-07
23	PF	BF	1.74E-03
24	SF	BF	2.09E-03
29	PF	GSF	1.59E-11
30	SF	GSF	5.70E-04
36	SF	PF	3.81E-09

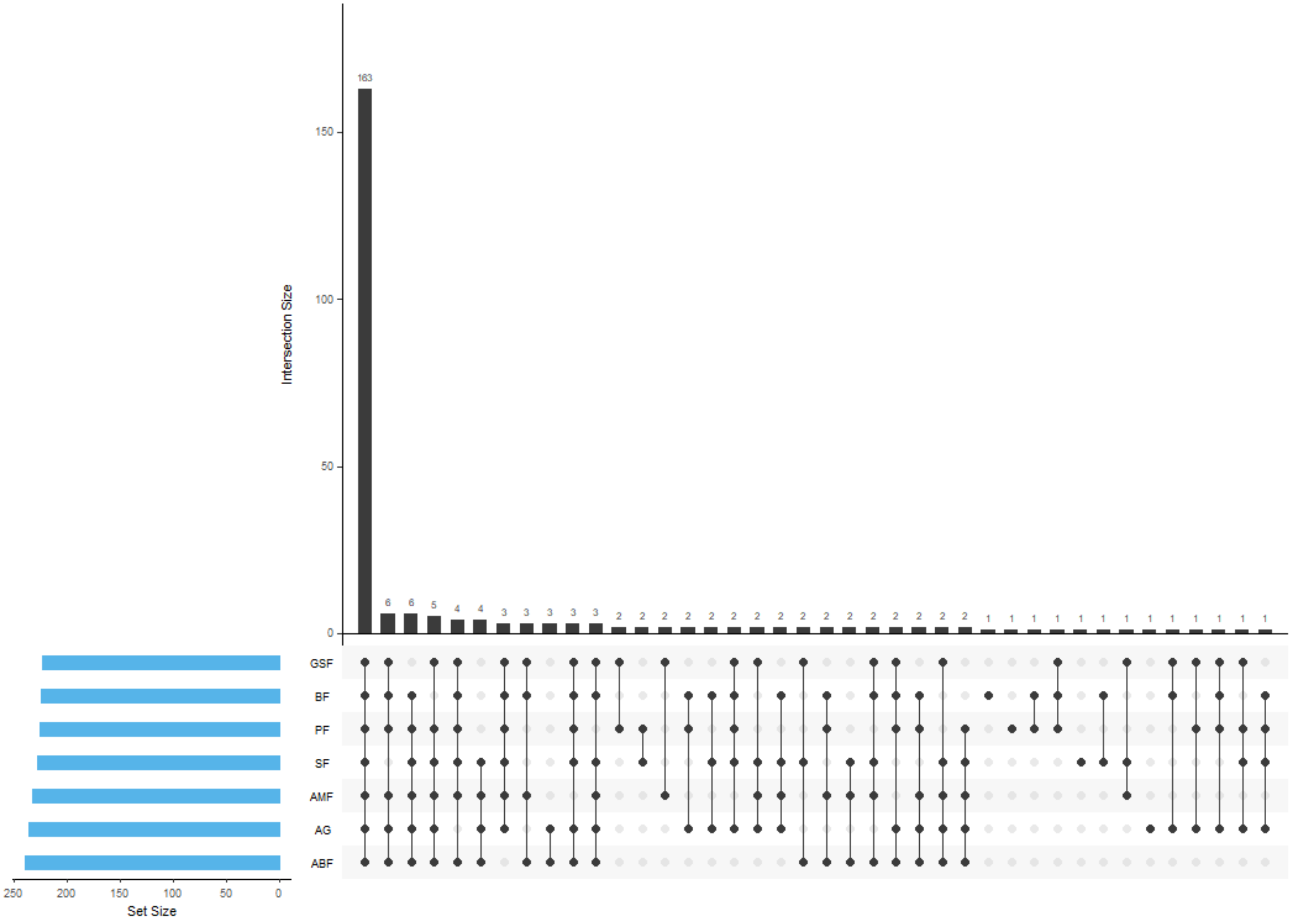

Fig. S2: Upset plot, showing the similarity of the microbial genera among the habitats. 163 genera were found in all the habitats

Habitat-specific representations of the microbiota with seasonal distribution

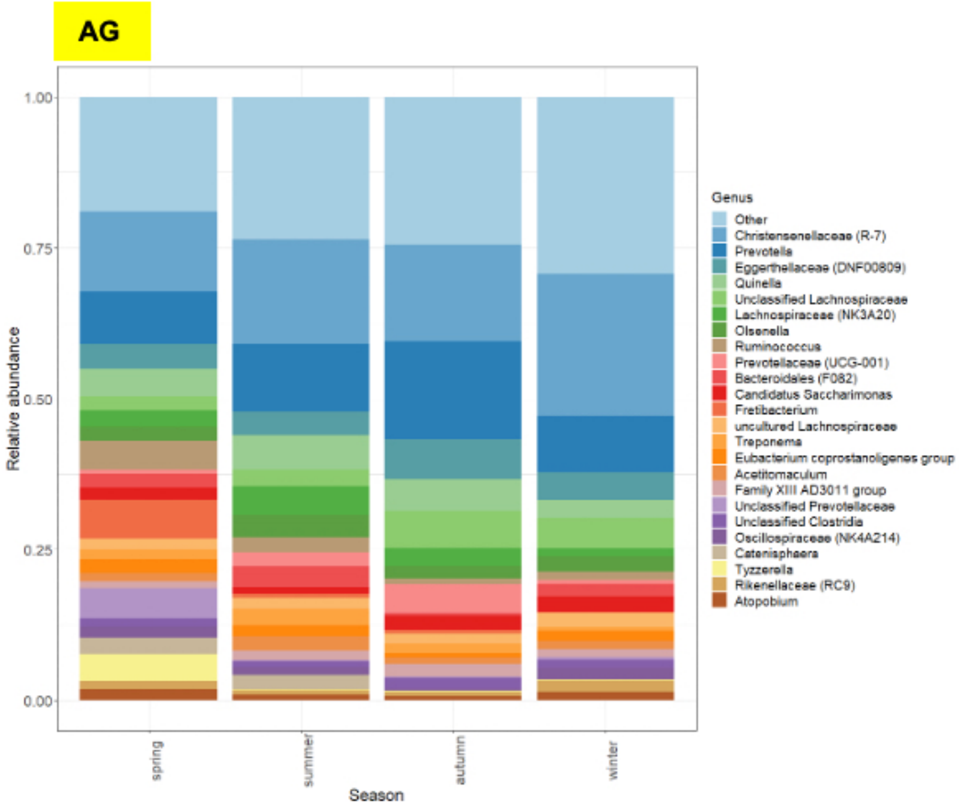

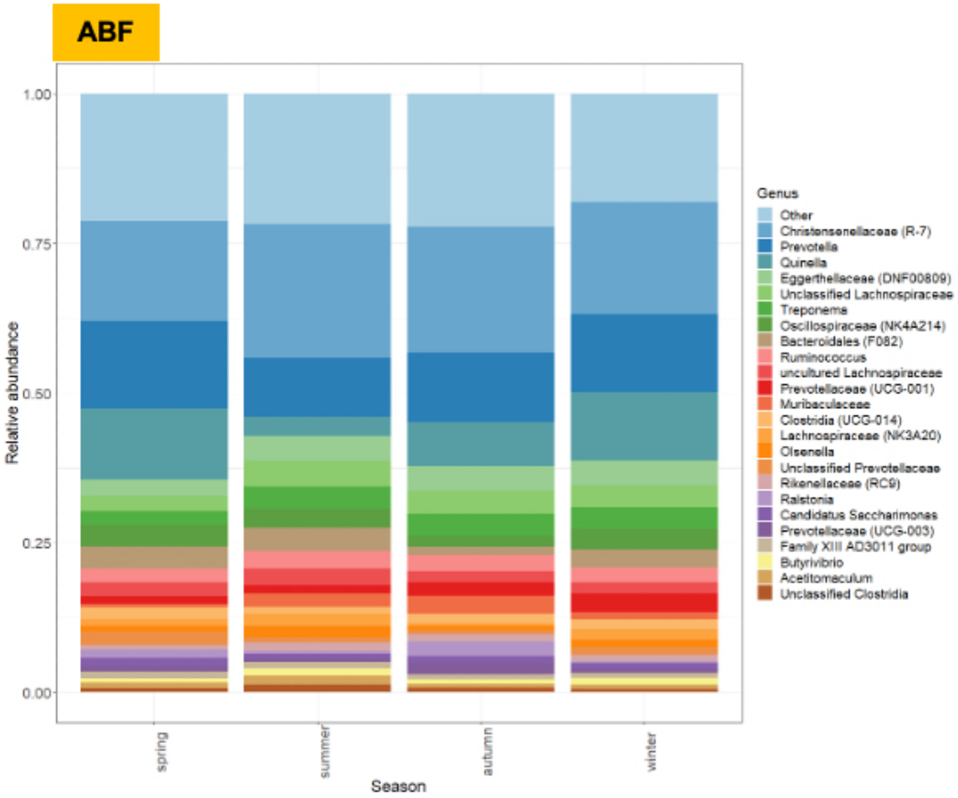

Fig. S3 A Overview of the seasonal distribution of bacterial genera across each habitat, top 24 genus level taxa, habitat AG and ABF

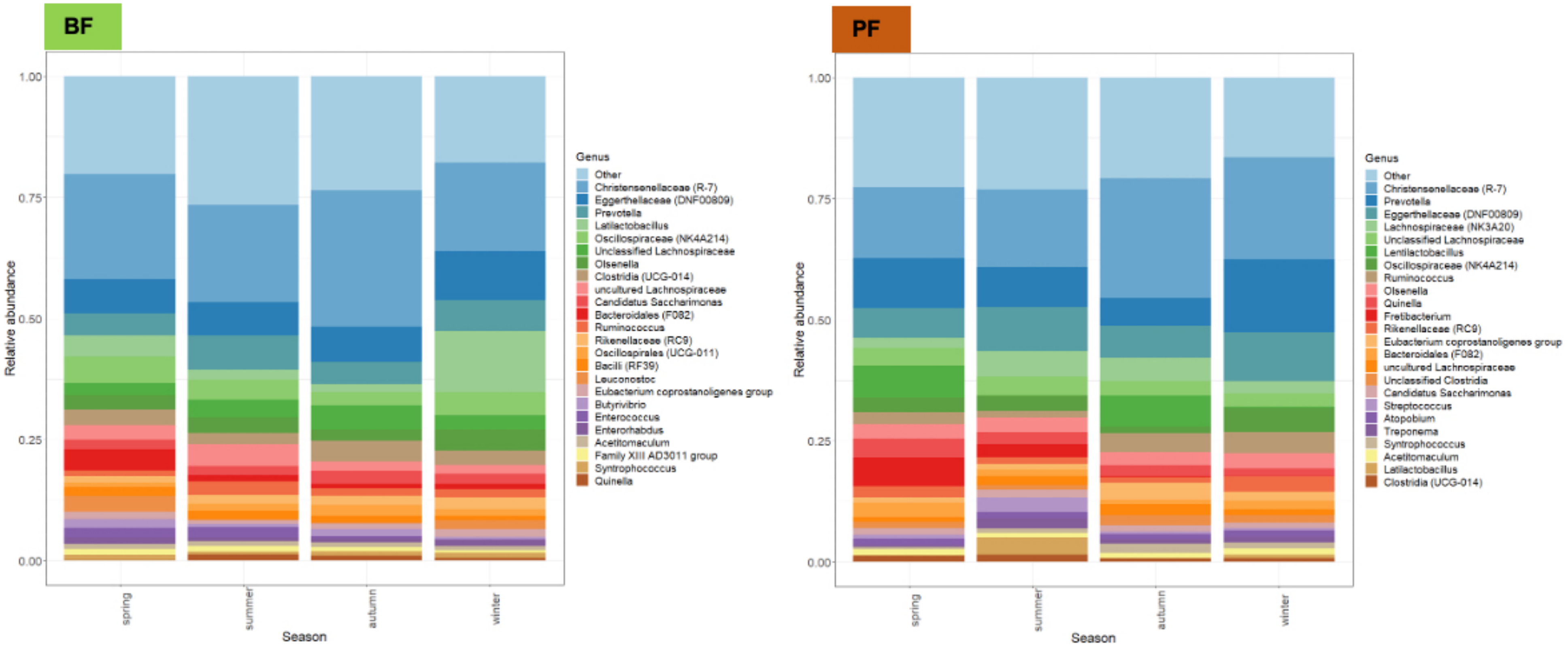

Fig. S3 B Overview of the seasonal distribution of bacterial genera across each habitat, top 24 genus level taxa, habitat BF and PF

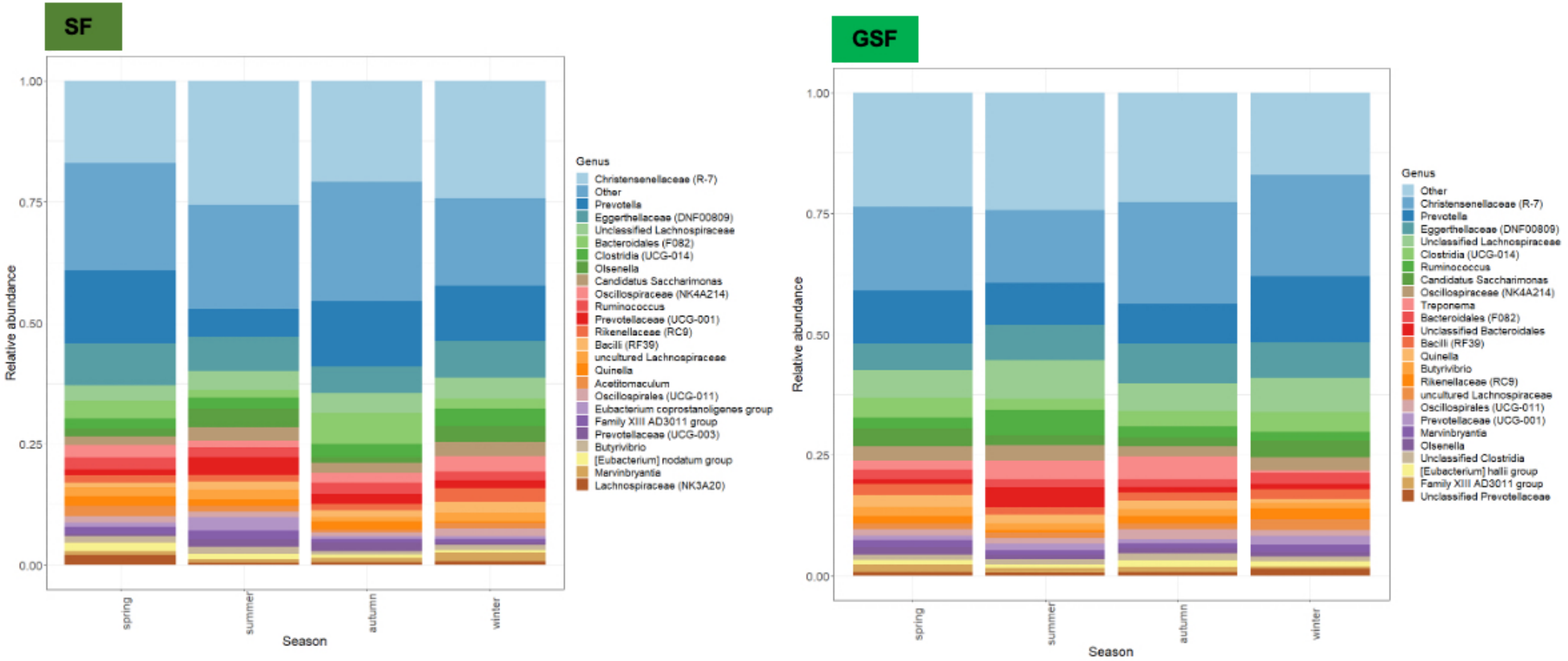

Fig. S3 C Overview of the seasonal distribution of bacterial genera across each habitat, top 24 genus level taxa, habitat SF and GSF

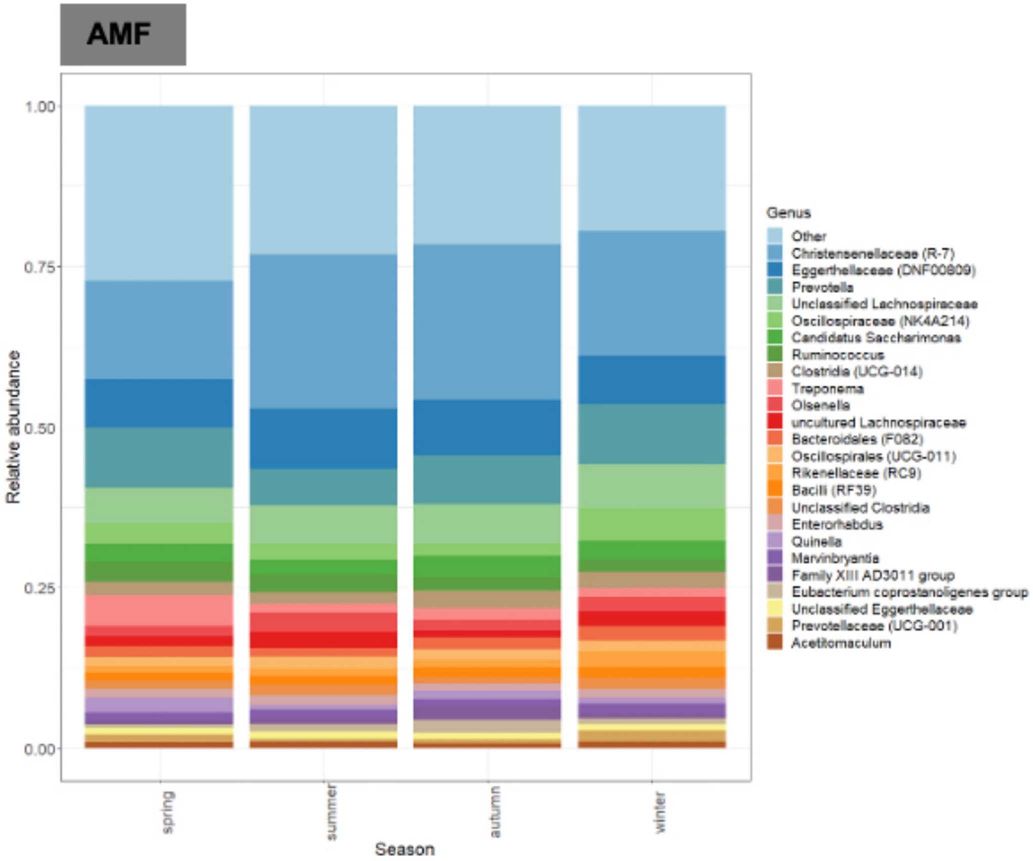

Fig. S3 D Overview of the seasonal distribution of bacterial genera across each habitat, top 24 genus level taxa, habitat AMF

Table S2 Distribution of frequencies of the 311 roe deer samples, n per unit

Habitat	AG	ABF	BF	PF	SF	GSF	AMF
	43	42	43	43	43	38	59

Season	spring	summer	autumn	winter
	74	75	80	82

Age class	adult	juvenile	subadult
	127	91	93

Gender	female	male
	151	160

Table S3 Local climate data of the study areas [3]

Habitat	Altitude above sea level [m]	Precipitation [mm]	Annual mean temperature [°C]	Mean temperature in growing season [°C]	Growing days	GPS coordinates
**Bavaria ∅**	560	970	7.0		145.1	
**Agriculture** (Lower Bavarian tertiary hills)	250-550	700-1025	7.5	12.5-13	145-165 (155)	48.404539 12.677600
**Agriculture-Beech-Forest** (Northern Franconian Plate)	150-450	650-749	7.8	12-12.5	145-175 (155)	50.048811 10.448186
**Beech Forest** (Bavarian colored sandstone Spessart)	150-550	500-1200	7.8	12-12.5	135-185 (153)	50.005782 9.379510
**Pine Forest** (Upper palatinate basin land)	350-650	600-900	7.4	12-12.5	125-165 (147)	49.328762 12.179269
**Spruce Forest** (Munich gravel plain)	450-750	700-1400	6.9	12-12.5	135-165 (148)	48.004758 11.435093
**Grassland-Spruce-Forest** (Allgäu molasse prealps)	650-1750	900-1100	5.4	11-11.5	68-155 (120)	47.568099 10.257149
**Alpine Mountain Forest** (Chiemgau alps)	450-1750	1025-1750	5.4	11-11.5	68-165 (129)	47.718425 12.605929

Table S4 Average content of crude nutrients and fermentation products in roe deer rumen content

	ø	sd	Min	Max	AG	ABF	BF	PF	SF	GSF	AMF
Crude protein [%] (CP)	23.60	5.61	10.31	38.74	26.80	22.99	22.71	19.75	26.78	22.95	23.26
Total lipids [%] (TL)	5.49	1.38	2.04	8.98	5.90	5.48	4.88	5.24	5.81	5.58	5.54
Crude ash [%] (CA)	7.69	1.58	3.63	11.95	7.16	8.39	7.60	6.38	8.50	7.76	7.99
Non-fibre carbohydrates [%] (NFC)	14.98	6.16	1.20	33.75	14.27	16.30	14.57	13.51	7.92	17.75	18.85
Neutral detergent fibre [%] (NDF)	47.93	8.50	28.60	69.93	44.95	45.59	50.20	54.48	51.03	45.97	44.31
Acid detergent fibre [%] (ADF)	33.34	7.24	17.80	52.05	28.83	30.92	35.66	36.98	33.94	35.44	32.20
Hemicellulose [%]	14.59	4.77	3.40	32.40	16.49	14.67	14.54	17.50	16.76	10.53	12.10
Cellulose [%]	19.33	4.41	8.00	31.30	16.72	18.68	21.88	21.29	17.52	20.35	19.09
Lignin [%]	13.88	4.35	3.77	26.70	12.11	12.25	14.04	15.69	15.29	15.09	13.11
Crude fibre [%] (CF)	27.80	6.99	13.16	49.87	26.22	24.08	28.87	30.56	29.22	29.29	26.81
Ammonia [mM]	18.98	8.71	2.21	45.42	20.06	25.22	18.64	20.50	13.90	19.63	13.80
Lactate [mM]	4.24	3.21	0.00	14.85	5.55	6.35	1.92	4.21	4.19	4.20	3.30
Acetic acid [mM]	60.50	16.27	22.22	102.59	60.42	67.08	59.93	57.76	53.60	59.24	64.03
Propionic acid [mM]	21.95	7.59	6.42	43.77	25.38	25.42	19.21	20.85	19.95	21.20	22.38
Butyric acid [mM]	12.63	5.28	3.38	26.92	13.25	13.26	9.72	10.69	10.30	15.37	15.09
Valeric acid [mM]	2.14	1.05	0.45	5.51	2.12	2.17	1.80	1.46	2.07	2.73	2.60
Isobutyric acid [mM]	0.51	0.25	0.06	1.25	0.57	0.69	0.42	0.45	0.37	0.60	0.50
Isovaleric acid [mM]	0.73	0.48	0.11	3.21	0.87	1.18	0.52	0.55	0.61	0.82	0.65

Table S5 Average content + standard deviation of crude nutrients and fermentation products in the rumen content of roe deer from the “Agriculture” habitat

Agriculture	*Ø spring*	sd spring	Ø summer	sd summer	Ø autumn	sd autumn	Ø winter	sd winter
**Crude protein [%]**	30.85	5.20	29.14	5.56	24.38	5.93	23.67	4.81
**Total lipids [%]**	7.17	1.60	5.99	1.76	6.01	2.11	4.91	1.04
**Crude ash [%]**	7.12	1.25	8.73	0.98	6.45	2.13	6.51	1.39
**Non-fibre carbohydrates [%]**	8.04	4.54	10.82	2.46	20.15	9.53	17.91	7.52
**Neutral detergent fibre [%]**	43.89	6.93	45.32	6.12	42.24	2.96	48.00	4.27
**Acid detergent fibre [%]**	28.57	6.10	28.43	3.95	26.27	7.56	31.52	3.71
**Hemicellulose [%]**	15.32	4.38	16.90	6.44	17.65	4.54	16.48	3.55
**Cellulose [%]**	14.82	4.27	18.21	2.21	16.39	4.96	17.77	1.77
**Lignin [%]**	13.75	3.31	10.22	5.38	9.89	3.52	13.75	2.50
**Crude fibre [%]**	26.19	6.30	24.11	4.61	24.88	7.67	28.96	4.04
**Ammonia [mM]**	24.47	9.86	22.58	6.25	20.58	9.09	13.35	13.35
**Lactate [mM]**	3.81	2.20	7.73	3.49	8.16	3.29	3.88	3.88
**Acetic acid [mM]**	53.28	16.16	63.57	15.39	67.29	17.58	60.64	60.64
**Propionic acid [mM]**	21.18	8.57	26.64	6.33	32.65	8.10	25.37	25.37
**Butyric acid [mM]**	9.65	5.02	12.37	3.81	18.37	5.03	14.96	14.96
**Valeric acid [mM]**	1.51	0.83	1.58	0.66	2.76	0.96	2.60	2.60
**Isobutyric acid [mM]**	0.60	0.17	0.65	0.25	0.59	0.41	0.45	0.45
**Isovaleric acid [mM]**	0.83	0.28	0.86	0.41	1.11	0.76	0.68	0.68

Table S6 Average content + standard deviation of crude nutrients and fermentation products in the rumen content of deer from the "Agriculture-Beech-Forest" habitat

Agriculture-Beech-Forest	***ø spring***	**sd spring**	**ø summer**	**sd summer**	**ø autumn**	**sd autumn**	**ø winter**	**sd winter**
**Crude protein [%]**	25.58	5.15	21.66	5.27	22.51	4.58	22.81	2.40
**Total lipids [%]**	6.26	1.52	5.31	1.14	5.29	1.16	5.16	1.02
**Crude ash [%]**	8.48	1.48	7.45	1.56	8.49	1.45	9.11	1.90
**Non-fibre carbohydrates [%]**	15.29	7.45	14.59	2.63	18.11	4.46	17.84	3.46
**Neutral detergent fibre [%]**	42.78	8.48	50.60	7.32	43.13	5.71	44.83	5.57
**Acid detergent fibre [%]**	30.01	8.14	32.99	6.98	28.57	5.46	31.59	3.95
**Hemicellulose [%]**	12.77	4.39	17.61	4.13	14.57	5.83	13.24	3.69
**Cellulose [%]**	18.62	4.07	20.88	4.81	15.94	5.05	18.76	3.11
**Lignin [%]**	11.39	4.53	12.11	4.43	12.62	3.21	12.83	3.83
**Crude fibre [%]**	21.69	5.46	27.74	7.73	22.13	3.11	23.96	4.07
**Ammonia [mM]**	24.15	6.85	28.38	8.48	20.42	10.48	27.01	12.31
**Lactate [mM]**	4.98	3.96	8.05	3.37	5.09	4.40	7.25	4.89
**Acetic acid [mM]**	62.57	16.49	70.04	16.78	69.88	13.83	65.00	18.87
**Propionic acid [mM]**	24.45	10.01	24.38	6.52	26.32	6.92	26.56	7.05
**Butyric acid [mM]**	11.52	3.17	12.11	4.03	16.29	4.51	12.41	4.02
**Valeric acid [mM]**	2.16	1.07	1.81	0.49	2.47	1.05	2.23	0.58
**Isobutyric acid [mM]**	0.59	0.16	0.82	0.31	0.69	0.27	0.61	0.32
**Isovaleric acid [mM]**	1.12	0.83	1.15	0.55	1.10	0.69	1.35	0.92

Table S7 Average content + standard deviation of crude nutrients and fermentation products in the rumen content of deer from the "Beech Forest" habitat

Beech Forest	***Ø spring***	***sd spring***	***Ø summer***	***sd summer***	***Ø autumn***	***sd autumn***	***Ø winter***	***sd winter***
Crude protein [%]	22.28	4.17	25.28	4.65	22.01	2.21	19.36	3.36
**Total lipids [%]**	4.85	0.62	5.24	1.38	5.22	1.21	3.58	0.77
**Crude ash [%]**	7.44	1.43	7.51	1.51	8.29	1.34	6.74	1.06
**Non-fibre carbohydrates [%]**	10.66	4.07	17.37	4.52	15.44	5.03	11.26	3.04
**Neutral detergent fibre [%]**	54.70	5.73	44.61	7.89	48.68	6.08	59.06	4.30
**Acid detergent fibre [%]**	38.81	5.10	33.01	8.34	34.06	4.53	40.34	4.13
**Hemicellulose [%]**	15.89	4.16	11.59	3.77	14.62	3.91	18.71	2.74
**Cellulose [%]**	23.88	3.89	20.14	4.03	21.21	2.79	24.09	2.03
**Lignin [%]**	14.94	2.28	13.47	5.11	12.85	3.00	16.26	2.27
**Crude fibre [%]**	31.41	3.42	27.81	6.70	27.00	7.57	31.53	2.52
**Ammonia [mM]**	22.81	9.89	19.10	7.35	17.67	5.68	15.48	3.45
**Lactate [mM]**	2.04	1.19	2.50	1.51	1.69	1.08	1.10	0.16
**Acetic acid [mM]**	58.17	11.03	61.65	16.89	62.83	13.69	52.67	9.12
**Propionic acid [mM]**	15.38	3.57	17.70	4.52	23.57	7.93	17.89	6.29
**Butyric acid [mM]**	9.11	1.30	9.18	3.07	10.48	4.73	9.83	5.01
**Valeric acid [mM]**	1.42	0.44	1.81	0.66	2.07	0.80	1.65	0.91
**Isobutyric acid [mM]**	0.57	0.10	0.39	0.14	0.44	0.21	0.29	0.18
**Isovaleric acid [mM]**	0.72	0.26	0.50	0.21	0.53	0.23	0.33	0.18

Table S8 Average content + standard deviation of crude nutrients and fermentation products in the rumen content of deer from the "Pine Forest" habitat

Pine Forest	*Ø spring*	*sd spring*	*Ø summer*	*sd summer*	*Ø autumn*	*sd autumn*	*Ø winter*	*sd winter*
Crude protein [%]	19.99	6.57	23.63	3.90	19.95	5.12	15.53	2.07
**Total lipids [%]**	4.92	1.05	6.15	1.39	5.34	0.96	4.57	1.10
**Crude ash [%]**	6.59	1.20	6.77	1.23	6.61	1.28	5.65	0.81
**Non-fibre carbohydrates [%]**	11.08	3.57	16.62	5.77	13.86	3.58	12.18	3.34
**Neutral detergent fibre [%]**	59.22	5.73	45.63	6.71	54.24	7.22	59.96	7.36
**Acid detergent fibre [%]**	39.46	6.97	30.08	4.98	36.60	6.76	42.30	6.91
**Hemicellulose [%]**	19.77	5.63	15.54	4.48	17.64	5.26	17.66	2.68
**Cellulose [%]**	22.86	3.92	17.45	3.19	20.16	5.54	24.82	4.28
**Lignin [%]**	16.60	4.20	12.63	2.81	16.44	5.12	17.48	3.17
**Crude fibre [%]**	32.51	7.39	25.78	4.43	28.62	5.87	35.32	6.05
**Ammonia [mM]**	20.72	10.55	20.96	6.81	20.28	7.81	20.03	6.58
**Lactate [mM]**	4.83	2.86	4.53	2.07	4.62	4.61	3.31	2.72
**Acetic acid [mM]**	56.39	9.21	55.20	16.36	70.27	12.35	50.94	16.05
**Propionic acid [mM]**	16.69	3.49	22.45	6.48	27.66	8.24	17.28	6.29
**Butyric acid [mM]**	8.83	2.44	11.31	5.31	12.54	2.61	10.13	5.70
**Valeric acid [mM]**	0.99	0.31	2.10	1.00	1.73	0.80	0.97	0.26
**Isobutyric acid [mM]**	0.37	0.14	0.60	0.26	0.46	0.14	0.34	0.12
**Isovaleric acid [mM]**	0.46	0.19	0.73	0.34	0.59	0.20	0.42	0.19

Table S9 Average content + standard deviation of crude nutrients and fermentation products in the rumen content of deer from the "Spruce Forest" habitat

Spruce Forest	***Ø spring***	***sd spring***	***Ø summer***	***sd summer***	***Ø autumn***	***sd autumn***	***Ø winter***	***sd winter***
Crude protein [%]	29.28	6.37	31.95	4.27	26.40	3.84	22.14	4.07
**Total lipids [%]**	6.07	1.34	7.32	1.23	5.87	0.93	4.71	0.78
**Crude ash [%]**	8.35	1.44	8.64	1.33	9.18	1.26	8.15	0.78
**Non-fibre carbohydrates [%]**	6.49	3.00	7.00	3.08	9.37	2.78	8.60	3.80
**Neutral detergent fibre [%]**	50.03	8.12	45.10	3.10	48.62	4.39	57.01	7.36
**Acid detergent fibre [%]**	31.37	7.27	31.00	2.43	32.19	4.16	39.08	7.06
**Hemicellulose [%]**	17.50	3.14	14.11	3.28	16.43	1.91	17.93	2.71
**Cellulose [%]**	15.36	4.75	18.57	2.39	18.03	1.78	18.66	2.95
**Lignin [%]**	16.01	3.88	12.43	3.64	14.17	3.08	17.42	3.59
**Crude fibre [%]**	27.12	5.47	26.27	3.25	28.03	2.64	33.56	5.78
**Ammonia [mM]**	17.27	10.27	15.19	2.43	12.59	6.14	11.31	8.55
**Lactate [mM]**	5.47	2.36	2.55	0.63	4.16	3.04	3.82	2.05
**Acetic acid [mM]**	52.60	10.72	62.90	12.84	68.52	8.79	39.56	11.68
**Propionic acid [mM]**	22.08	6.69	24.20	5.53	22.69	3.78	13.95	5.68
**Butyric acid [mM]**	10.98	2.77	10.95	2.35	13.65	3.26	7.22	2.86
**Valeric acid [mM]**	1.76	0.81	2.09	0.36	3.23	0.76	1.48	1.12
**Isobutyric acid [mM]**	0.42	0.15	0.42	0.14	0.40	0.14	0.28	0.09
**Isovaleric acid [mM]**	0.70	0.41	0.57	0.22	0.90	0.44	0.36	0.14

Table S10 Average content + standard deviation of crude nutrients and fermentation products in the rumen content of deer from the "Grassland-Spruce-Forest" habitat

Grassland-Spruce-Forest	*Ø spring*	*sd spring*	*Ø summer*	*sd summer*	*Ø autumn*	*sd autumn*	*Ø winter*	*sd winter*
Crude protein [%]	26.17	5.24	23.39	3.83	21.79	2.11	20.97	3.81
**Total lipids [%]**	5.97	1.34	6.01	1.13	5.55	1.23	5.04	0.71
**Crude ash [%]**	7.77	0.73	8.38	1.03	7.57	1.43	7.65	0.86
**Non-fibre carbohydrates [%]**	19.15	5.96	17.98	1.83	18.36	5.57	15.75	3.70
**Neutral detergent fibre [%]**	40.93	8.00	44.24	6.27	46.73	6.80	50.58	6.67
**Acid detergent fibre [%]**	32.03	5.95	34.68	5.28	35.75	5.65	38.58	5.45
**Hemicellulose [%]**	8.90	3.36	9.56	1.75	10.97	3.33	12.00	2.49
**Cellulose [%]**	19.01	4.18	21.14	4.55	20.26	2.43	21.29	4.34
**Lignin [%]**	13.02	3.91	13.54	1.79	15.49	3.98	17.29	3.09
**Crude fibre [%]**	23.91	4.03	27.98	4.00	34.66	10.32	29.45	4.86
**Ammonia [mM]**	21.30	8.40	26.34	8.44	19.64	7.44	15.21	5.52
**Lactate [mM]**	4.69	2.95	3.02	1.83	3.16	1.72	5.45	3.23
**Acetic acid [mM]**	58.70	13.41	62.92	12.47	69.62	13.11	47.34	12.29
**Propionic acid [mM]**	19.65	5.37	23.59	6.23	28.01	5.97	15.11	4.68
**Butyric acid [mM]**	14.83	5.61	16.77	4.97	20.13	4.66	10.34	3.64
**Valeric acid [mM]**	2.75	1.37	3.47	1.35	3.13	1.22	2.01	0.78
**Isobutyric acid [mM]**	0.65	0.27	0.74	0.27	0.60	0.27	0.47	0.15
**Isovaleric acid [mM]**	0.90	0.39	0.96	0.40	0.82	0.42	0.66	0.23

Table S11 Average content + standard deviation of crude nutrients and fermentation products in the rumen content of deer from the "Alpine Mountain Forest" habitat

Alpine Mountain Forest	*Ø spring*	*sd spring*	*Ø summer*	*sd summer*	*Ø autumn*	*sd autumn*	*Ø winter*	*sd winter*
Crude protein [%]	26.21	5.33	24.51	3.62	22.83	2.57	19.46	3.95
**Total lipids [%]**	5.28	1.26	6.02	1.02	5.94	0.84	4.97	0.86
**Crude ash [%]**	7.81	0.96	8.56	1.80	8.12	1.25	7.46	1.19
**Non-fibre carbohydrates [%]**	17.43	4.48	19.95	3.39	20.23	3.39	17.89	4.18
**Neutral detergent fibre [%]**	43.27	7.47	40.95	5.33	42.66	5.65	50.23	6.58
**Acid detergent fibre [%]**	30.80	5.96	28.58	5.68	31.66	4.84	37.74	6.58
**Hemicellulose [%]**	12.47	5.02	12.37	2.61	11.01	2.62	12.49	2.89
**Cellulose [%]**	17.77	3.37	17.56	2.69	19.73	3.03	21.36	3.15
**Lignin [%]**	13.03	4.59	11.02	3.83	11.93	2.58	16.38	4.73
**Crude fibre [%]**	23.82	4.22	24.42	6.11	29.50	10.50	29.86	5.91
Ammonia [mM]	11.94	8.05	13.25	5.51	17.15	3.51	12.33	4.16
Lactate [mM]	3.08	2.95	3.46	2.00	3.38	1.10	3.34	2.59
Acetic acid [mM]	69.76	12.91	70.63	13.25	62.70	13.96	52.95	16.71
Propionic acid [mM]	23.29	5.21	21.82	5.71	26.01	5.62	18.64	6.91
Butyric acid [mM]	16.61	4.25	15.03	4.37	17.01	3.91	11.84	4.88
Valeric acid [mM]	2.98	0.78	2.55	0.68	2.94	0.88	1.97	0.71
Isobutyric acid [mM]	0.57	0.24	0.57	0.21	0.41	0.14	0.43	0.17
Isovaleric acid [mM]	0.66	0.32	0.84	0.31	0.57	0.18	0.51	0.25

Table S12 Relative abundance of bacterial phyla per habitat

Phylum	AG	ABF	BF	PF	SF	GSF	AMF
Acidobacteriota	2.56E-05	1.71E-05	0	0	3.95E-05	0	2.59E-05
Actinobacteriota	0.119948837	0.083878049	0.155162791	0.158293023	0.143816279	0.132986842	0.1658
Bacteroidota	0.20927907	0.238060976	0.121604651	0.174730233	0.229260465	0.192157895	0.157412069
Campylobacterota	9.30E-06	0.000243902	9.30E-06	4.65E-06	0.000130233	0.000694737	3.79E-05
Cyanobacteria	0.000393023	0.000507317	0.000448837	0.000151163	0.000397674	3.00E-04	0.00022931
Desulfobacterota	1.40E-05	0.000109756	5.12E-05	4.65E-06	9.53E-05	5.26E-06	0
Fibrobacterota	3.26E-05	2.44E-06	4.19E-05	0.000106977	1.63E-05	2.63E-05	3.45E-06
Firmicutes	0.599504651	0.611429268	0.68517907	0.615139535	0.582497674	0.608497368	0.612362069
Patescibacteria	0.020962791	0.010668293	0.022197674	0.013504651	0.024606977	0.028534211	0.028581034
Proteobacteria	0.01312093	0.014529268	0.007395349	0.005295349	0.007481395	0.007021053	0.010062069
Spirochaetota	0.015690698	0.033853659	0.005962791	0.012667442	0.009913953	0.026555263	0.023091379
Synergistota	0.020990698	0.006639024	0.001502326	0.020076744	0.001653488	0.003176316	0.002353448
Verrucomicrobiota	2.79E-05	6.10E-05	0.000444186	2.56E-05	9.07E-05	4.47E-05	4.14E-05

Table S13 Mean relative abundance of bacterial genera per habitat

Genus	AG	ABF	BF	PF	SF	GSF	AMF
Absconditabacteriales (SR1)	9.53E-05	0.00027561	3.26E-05	0.000132558	0.00047907	0.000671053	0.00047931
Acetitomaculum	0.015190698	0.00904878	0.009753488	0.011148837	0.012344186	0.007239474	0.009587931
Acholeplasma	6.98E-06	9.76E-05	0	4.42E-05	6.98E-06	0	0
Acidiphilium	9.30E-06	3.41E-05	0	0	1.16E-05	5.26E-06	7.41E-05
Actinomyces	0.00015814	7.80E-05	0.00022093	0.000167442	0.000425581	0.000184211	0.000234483
Actinomycetospora	9.30E-06	0	0	0	2.79E-05	4.47E-05	3.97E-05
Aerococcus	2.79E-05	8.05E-05	3.72E-05	0	3.95E-05	7.89E-06	0
Aeromicrobium	2.33E-06	9.76E-06	1.63E-05	6.98E-06	0	0	5.17E-06
Agreia	4.19E-05	0	4.42E-05	4.65E-06	3.26E-05	5.26E-06	0
Alloprevotella	0.000111628	3.17E-05	1.16E-05	0	3.72E-05	5.00E-05	5.86E-05
Allorhizobium-Neorhizobium-Pararhizobium-Rhizobium	0.000418605	0.000190244	0.000132558	9.53E-05	9.07E-05	7.37E-05	6.72E-05
Aminicenantales	2.56E-05	1.71E-05	0	0	3.95E-05	0	2.59E-05
Amnibacterium	2.33E-06	0	1.63E-05	0	2.09E-05	2.63E-06	5.17E-06
Amnipila	5.58E-05	0.000553659	0.000253488	0.00014186	0.000153488	0.000610526	0.000215517
Anaerobiospirillum	0	0	0	3.49E-05	0	0	0
Anaerofustis	2.79E-05	0.000158537	0.000202326	0.000102326	5.81E-05	8.16E-05	0.000106897
Anaerostipes	0	0	0	0.000151163	0	5.26E-06	0
Anaerovorax	0.000437209	0.000621951	0.000572093	0.00057907	0.000693023	0.00075	0.000701724
ASF356	0	2.44E-06	0	0	3.49E-05	0	3.45E-06
Atopobium	0.012467442	0.006995122	0.007327907	0.012146512	0.00715814	0.006234211	0.008681034
Aureimonas	1.00E-04	4.88E-05	7.21E-05	0.000144186	4.88E-05	0.000121053	6.21E-05
Bacilli (RF39)	0.007497674	0.006812195	0.015427907	0.005709302	0.016725581	0.018563158	0.014503448
Bacteroidales (F082)	0.020393023	0.029473171	0.017572093	0.017567442	0.033293023	0.019121053	0.01782931
Bacteroidales (P-251-O5)	3.02E-05	0.000885366	0.000190698	0.000523256	0.000809302	0.000768421	0.000653448
Bacteroidales (P-2534-18B5)	0.000686047	0.002229268	0.001395349	0.002406977	0.001225581	0.000607895	0.00097069

Bacteroidales (PEH15)	0	9.76E-06	0	0	0	2.63E-06	0
Bacteroidales (RH-AAJ90H05)	0.000465116	0.000304878	0.000290698	0.000139535	0.000211628	7.11E-05	1.00E-04
Bacteroidales BS11 Gut Group	4.88E-05	3.66E-05	3.26E-05	9.30E-06	7.91E-05	5.26E-06	2.07E-05
Bacteroidales RF16 Group	0.00094186	0.000714634	4.42E-05	0.001323256	0.000106977	0.000278947	0.000555172
Bacteroidales UCG-001	0	2.44E-06	0	0	0	0	6.90E-06
Bacteroides	4.65E-06	3.41E-05	4.65E-06	9.30E-06	2.33E-06	2.63E-06	0
Beijerinckiaceae 1174-901-12	0.000130233	0.000102439	0.000165116	0.000151163	0.00012093	0.000252632	0.001160345
Bibersteinia	0	7.32E-06	4.65E-06	1.63E-05	4.88E-05	1.32E-05	5.00E-05
Bifidobacterium	2.56E-05	4.88E-06	6.98E-06	0.002502326	0	0.007634211	0
Blautia	0.001709302	0.001843902	0.006044186	0.002834884	0.004786047	0.003828947	0.0022
Brachybacterium	0	4.39E-05	0	0	9.30E-06	1.05E-05	0
Brachyspira	0	1.22E-05	0	0	1.16E-05	2.11E-05	1.72E-06
Bradymonadales	0	6.83E-05	2.09E-05	0	9.53E-05	2.63E-06	0
Bradyrhizobium	0.000123256	6.10E-05	4.42E-05	2.33E-06	6.28E-05	3.68E-05	3.97E-05
Brevibacterium	4.65E-06	3.90E-05	0	0	0.000290698	0	1.21E-05
Brevundimonas	6.98E-06	4.63E-05	4.65E-06	1.16E-05	1.40E-05	0	1.90E-05
Brochothrix	0	1.95E-05	0.000602326	0	0	5.26E-06	1.03E-05
Burkholderia-Caballeronia-Paraburkholderia	0.009604651	0.00027561	0.002769767	5.12E-05	0.004434884	0.000123684	0.000284483
Butyricicoccaceae (UCG-008)	0	0	0	5.12E-05	6.05E-05	0	0
Butyrivibrio	0.004711628	0.00967561	0.011313953	0.005290698	0.010834884	0.01475	0.00825
Caldibacillus	0	0	0	0	7.44E-05	0.000107895	4.14E-05
Candidatus Arthromitus	0.000606977	0.000631707	0.000332558	0.000439535	0.000344186	0.000363158	0.000384483
Candidatus Saccharimonas	0.020867442	0.010392683	0.022165116	0.013372093	0.024127907	0.027863158	0.028101724
Candidatus Soleaferrea	0.000223256	0.000168293	0.00142093	0.001653488	0.00107907	0.002647368	0.002901724
Carnobacterium	6.98E-06	8.05E-05	0.00572093	2.33E-06	0	0.003463158	2.07E-05
Catenisphaera	0.012846512	0.000602439	0.00104186	0.004862791	0.000644186	0.000405263	0.000806897
Christensenellaceae (R-7)	0.175893023	0.197134146	0.226988372	0.191727907	0.218516279	0.190852632	0.205125862
Clavibacter	3.02E-05	0	9.30E-06	0	2.33E-06	0	0

Clostridia (UCG-014)	0.011144186	0.0152	0.031830233	0.010795349	0.027683721	0.035631579	0.023043103
Clostridia vadin BB60 Group	0	2.44E-06	2.79E-05	0	2.33E-06	4.21E-05	3.45E-06
Clostridium Sensu Stricto 1	6.74E-05	2.44E-05	0.003523256	6.98E-06	0	3.95E-05	3.45E-06
Clostridium Sensu Stricto 2	0	0	0.000123256	0	0	0	0
Colidextribacter	0.000348837	0.000902439	0.001018605	0.000432558	0.000348837	0.000931579	0.000343103
Coprococcus	0.000351163	0.000960976	3.26E-05	0.00062093	0.000209302	0.000344737	0.000268966
Coriobacteriaceae (UCG-002)	0.000223256	0.000209756	0.000137209	0.000123256	0.000102326	0.000194737	9.14E-05
Coriobacteriales	0.000376744	0.000490244	0.001602326	0.000274419	0.000802326	0.001642105	0.001615517
Corynebacterium	2.56E-05	0.00044878	6.05E-05	3.49E-05	0.000525581	0	0.000194828
Coxiella	0	0	0	8.60E-05	0	5.26E-06	0
Curtobacterium	3.26E-05	9.76E-06	6.98E-06	6.98E-06	2.33E-05	2.63E-06	8.62E-06
Cutibacterium	0.000204651	0.001819512	0.000281395	0.000181395	0.00014186	6.32E-05	0.000863793
Defluviitaleaceae (UCG-011)	0.000262791	0.000714634	0.000346512	0.000193023	0.000513953	0.000342105	0.000175862
Desulfovibrio	1.40E-05	4.15E-05	3.02E-05	4.65E-06	0	2.63E-06	0
Devosia	9.30E-06	2.44E-06	1.63E-05	2.09E-05	1.86E-05	5.26E-06	1.55E-05
Eggerthellaceae (DNF00809)	0.047811628	0.037707317	0.075076744	0.08164186	0.072416279	0.071484211	0.083691379
Enhydrobacter	6.98E-06	2.20E-05	0	2.33E-06	6.98E-06	0	6.90E-06
Enorma	0	0	0	0.000167442	0.000111628	0	0
Enterobacter	6.98E-06	6.59E-05	0	0	0	0	0
Enterococcus	9.07E-05	2.68E-05	0.011639535	0.002372093	3.72E-05	6.84E-05	6.90E-05
Enterorhabdus	0.005495349	0.004653659	0.010044186	0.008030233	0.008872093	0.008592105	0.013427586
Erwinia	4.19E-05	2.44E-06	0	0	2.33E-06	1.58E-05	0
Erysipelatoclostridiaceae (UCG-004)	5.35E-05	1.71E-05	0	0	2.33E-06	0	8.62E-06
Erysipelothrix	0	5.61E-05	0	0	0	0	0
Erysipelotrichaceae (UCG-002)	0.000683721	0.000126829	0	1.16E-05	0	0	0
Erysipelotrichaceae (UCG-007)	0.000795349	0.000607317	0.000223256	0.000388372	0.00102093	0.000505263	0.000424138
Erysipelotrichaceae (UCG-009)	0.011176744	0.001092683	0.000348837	0.000493023	0.000423256	0.000373684	0.000174138
Escherichia-Shigella	5.58E-05	6.10E-05	3.72E-05	1.16E-05	3.95E-05	2.11E-05	3.28E-05

Eubacterium Coprostanoligenes Group	0.016448837	0.008219512	0.011986047	0.018116279	0.01062093	0.004536842	0.011001724
Family Xiii AD3011 Group	0.014930233	0.009529268	0.00942093	0.01034186	0.010916279	0.009652632	0.011389655
Family Xiii UCG-001	0.000569767	0.001019512	0.001495349	0.00095814	0.001625581	0.001892105	0.000839655
FD2005	0.000239535	0.000236585	3.02E-05	0.000153488	0	0	1.72E-06
Fibrobacter	3.26E-05	2.44E-06	4.19E-05	0.000106977	1.63E-05	2.63E-05	3.45E-06
Fretibacterium	0.020946512	0.006639024	0.001502326	0.020069767	0.001648837	0.003176316	0.002343103
Friedmanniella	6.98E-06	2.44E-06	0	4.65E-06	2.33E-06	4.21E-05	1.21E-05
Frigoribacterium	0.000106977	3.66E-05	1.16E-05	1.86E-05	4.65E-06	7.89E-06	0
Frondihabitans	2.33E-06	7.32E-06	1.16E-05	6.98E-06	2.33E-06	1.32E-05	0
Gastranaerophilales	0.000393023	0.000507317	0.000448837	0.000151163	0.000397674	3.00E-04	0.00022931
Gluconobacter	0	0	3.26E-05	9.30E-06	0	2.63E-06	0
Gordonibacter	0.000209302	6.83E-05	7.44E-05	0.000130233	0.000134884	0.000192105	0.000203448
Helicobacter	9.30E-06	0.000243902	9.30E-06	4.65E-06	0.000130233	0.000694737	3.79E-05
Herbinix	2.33E-05	9.76E-06	0	0	0	0	0
Howardella	0.008395349	0.003407317	0.004918605	0.00472093	0.0077	0.006863158	0.007718966
Incertae Sedis	0.00197907	0.002207317	0.001104651	0.001211628	0.000606977	0.000563158	0.000374138
Jatrophihabitans	2.33E-06	0	0	0	2.33E-06	1.58E-05	2.76E-05
Kocuria	0	2.20E-05	4.65E-06	1.40E-05	0	0	1.72E-06
Komagataeibacter	9.30E-06	0	1.86E-05	0	2.79E-05	0	0
Lachnoclostridium	2.33E-06	0	2.33E-06	3.26E-05	2.33E-06	0	0
Lachnospiraceae (NK3A20)	0.028397674	0.013485366	0.003472093	0.036844186	0.010367442	0.003378947	0.002794828
Lachnospiraceae (UCG-001)	1.16E-05	2.93E-05	1.40E-05	1.16E-05	1.40E-05	2.37E-05	0.00012931
Lachnospiraceae (UCG-002)	0.004172093	0.00207561	0.003348837	0.004790698	0.002613953	0.001655263	0.002737931
Lachnospiraceae (UCG-008)	0.000506977	0.000909756	0.001402326	0.000516279	0.001337209	0.000836842	0.001715517
Lachnospiraceae AC2044 Group	6.51E-05	0.000563415	0.000116279	0.000316279	0.00027907	0.000189474	0.000227586
Lachnospiraceae FE2018 Group	0.001751163	0.001253659	0.00132093	0.000806977	0.000806977	0.000668421	0.000701724
Lachnospiraceae ND3007 Group	0.001202326	0.000787805	0.000106977	0.000623256	0.000255814	8.42E-05	0.000162069
Lachnospiraceae NK4A136 Group	0.002188372	0.002329268	0.001130233	0.002495349	0.001506977	0.003815789	0.002672414

Lachnospiraceae XPB1014 Group	0.000846512	0.000487805	0	0.000362791	0	1.84E-05	0
Lacticaseibacillus	0	2.44E-06	0	0.002616279	1.40E-05	0	0
Lactiplantibacillus	9.30E-06	0	0.00104186	0.000872093	1.86E-05	2.63E-06	0
Lactobacillus	0.000323256	3.00E-04	0.000118605	8.14E-05	0.000123256	0.000186842	0.000287931
Lactococcus	1.16E-05	9.51E-05	0.005416279	4.65E-06	9.30E-06	0	2.41E-05
Latilactobacillus	2.33E-05	0.000102439	0.041125581	0.012211628	0	5.26E-06	3.45E-05
Lawsonella	6.98E-05	0.000373171	8.14E-05	6.51E-05	1.86E-05	0	0.000118966
Lentilactobacillus	4.65E-06	8.29E-05	0.003074419	0.029169767	2.09E-05	7.89E-06	2.41E-05
Leuconostoc	4.65E-05	0.000178049	0.010009302	0.000455814	2.33E-06	5.00E-05	5.17E-06
Levilactobacillus	4.65E-06	0	0.000144186	6.28E-05	0	0	0
Ligilactobacillus	0.001009302	0.001065854	0.000711628	0.000744186	0.00095814	0.000557895	0.000777586
Limosilactobacillus	3.26E-05	7.32E-06	6.98E-06	2.09E-05	0	2.63E-06	2.59E-05
Liquorilactobacillus	0	0	0	0	0.000102326	0	0
Marvinbryantia	0.008648837	0.0084	0.008472093	0.006690698	0.010855814	0.011981579	0.012608621
Massilia	4.65E-06	9.76E-06	2.33E-06	4.88E-05	1.16E-05	1.05E-05	1.55E-05
Megamonas	1.16E-05	0	4.88E-05	0	0	2.63E-06	0
Mesorhizobium	2.33E-05	6.83E-05	2.33E-05	2.33E-06	4.65E-06	1.05E-05	5.00E-05
Methylobacterium-Methylorubrum	0.000162791	5.61E-05	0.000109302	0.000148837	9.07E-05	0.000110526	9.83E-05
Microbacterium	0.000202326	0	4.65E-06	4.65E-06	0	7.89E-06	0
Micrococcus	6.98E-06	7.32E-06	0	0	2.33E-05	7.89E-06	3.45E-06
Microlunatus	0	4.88E-06	4.65E-06	0	1.16E-05	5.26E-06	1.72E-06
Mogibacterium	0.000704651	0.000287805	0.000746512	0.000816279	0.000606977	0.000860526	0.000689655
Monoglobus	0.001253488	0.006280488	0.00552093	0.002723256	0.003674419	0.00775	0.006782759
Muribaculaceae	0.009374419	0.018487805	0.003248837	0.0085	0.006381395	0.004294737	0.007863793
Mycobacterium	0	7.32E-06	2.33E-05	2.33E-06	9.30E-06	5.26E-06	6.90E-06
Nakamurella	4.65E-06	1.22E-05	9.30E-06	0	2.33E-06	7.37E-05	3.79E-05
Oenococcus	0	7.32E-06	0	0	0.000174419	7.89E-06	0
Olsenella	0.02594186	0.012385366	0.030839535	0.02942093	0.025009302	0.0117	0.021484483

Oribacterium	0	9.76E-06	0	4.65E-06	0	0	0
Oscillibacter	0.002037209	0.003719512	0.001502326	0.000416279	0.002969767	0.007321053	0.007865517
Oscillospira	0.00072093	0.002141463	0.001393023	0.000432558	0.005388372	0.002521053	0.00462931
Oscillospiraceae	9.30E-06	9.76E-06	1.16E-05	2.09E-05	0	2.63E-06	1.90E-05
Oscillospiraceae (NK4A214)	0.013783721	0.029819512	0.040762791	0.0317	0.024	0.026860526	0.031668966
Oscillospiraceae (UCG-002)	0.001448837	0.00487561	0.000793023	0.000653488	0.001990698	0.001986842	0.00402931
Oscillospiraceae (UCG-005)	0.001811628	0.001826829	0.004374419	0.00312093	0.002890698	0.002215789	0.003594828
Oscillospiraceae (UCG-007)	2.33E-06	2.20E-05	6.98E-06	4.65E-06	1.16E-05	5.00E-05	1.03E-05
Oscillospiraceae-V9D2013 Group	2.33E-06	0	1.86E-05	7.21E-05	1.86E-05	4.74E-05	9.66E-05
Oscillospirales	2.33E-05	0	0	0	0	4.74E-05	4.31E-05
Oscillospirales (UCG-010)	0.000248837	0.000853659	0.000539535	0.000255814	0.000332558	0.000484211	0.000460345
Oscillospirales (UCG-011)	0.008437209	0.006185366	0.016248837	0.009825581	0.012502326	0.014728947	0.01582069
Oxalobacter	6.28E-05	4.63E-05	3.26E-05	4.19E-05	0.000123256	9.47E-05	8.10E-05
Papillibacter	2.33E-06	0	0.000183721	2.09E-05	0.000113953	1.58E-05	3.45E-06
Paracoccus	2.33E-06	6.34E-05	2.33E-06	0	0	0	0
Paucilactobacillus	0	4.88E-06	4.65E-06	3.26E-05	0	7.89E-06	1.72E-06
Pediococcus	0	0	0.000137209	3.49E-05	0	0	0
Phoenicibacter	0	0	7.44E-05	0	6.98E-05	0	0
Plantibacter	5.81E-05	4.88E-06	2.33E-06	1.86E-05	0	7.89E-06	1.90E-05
Prevotella	0.112051163	0.122763415	0.056660465	0.100083721	0.117983721	0.105023684	0.079468966
Prevotella (9)	0	2.93E-05	0	0	2.33E-06	0	5.17E-06
Prevotellaceae (NK3B31)	0.001086047	0.001558537	0.00135814	0.000860465	0.003411628	0.000847368	0.001584483
Prevotellaceae (UCG-001)	0.020223256	0.020217073	0.006644186	0.008616279	0.019946512	0.012568421	0.010467241
Prevotellaceae (UCG-003)	0.009186047	0.01037561	0.003474419	0.003793023	0.01062093	0.004960526	0.008310345
Prevotellaceae (UCG-004)	3.72E-05	0.000131707	0.000234884	4.88E-05	0.000790698	0	5.17E-06
Propionibacterium	0.000213953	0.00012439	9.07E-05	0.000162791	0.000190698	0.000134211	0.000131034
Pseudobutyrivibrio	0.000248837	7.00E-04	0.002483721	0.000546512	0.001146512	0.001271053	0.000853448
Pseudochrobactrum	0	2.44E-06	6.98E-06	1.40E-05	1.40E-05	0	4.31E-05

Pseudoclavibacter	3.72E-05	7.32E-06	0	0	0	0	0
Pseudomonas	8.84E-05	4.39E-05	4.88E-05	6.98E-06	4.19E-05	1.58E-05	3.28E-05
Psychrobacter	1.16E-05	7.07E-05	0	0	0	0	1.72E-05
Pyramidobacter	4.42E-05	0	0	6.98E-06	4.65E-06	0	1.03E-05
Quinella	0.04455814	0.083763415	0.008832558	0.025327907	0.01294186	0.016489474	0.013343103
Ralstonia	0.000113953	0.011546341	0.002216279	0.002772093	8.60E-05	0.004278947	0.005031034
Raoultibacter	0.003862791	0.002607317	0.002293023	0.002069767	0.003069767	0.002205263	0.002010345
Reyranella	0	4.39E-05	2.33E-06	0	0	3.16E-05	2.07E-05
Rhodococcus	6.74E-05	1.46E-05	4.65E-06	2.33E-06	0	0	0
Rikenellaceae (RC9)	0.012602326	0.011882927	0.017404651	0.020460465	0.018493023	0.015455263	0.014813793
Romboutsia	3.02E-05	1.95E-05	1.16E-05	3.49E-05	6.98E-06	2.89E-05	2.76E-05
Roseburia	0.000932558	0.001463415	0.004111628	0.003518605	0.001986047	0.003773684	0.004591379
Roseiarcus	9.30E-06	4.88E-06	2.33E-06	9.30E-06	1.63E-05	0	1.72E-06
Ruminiclostridium	0	9.76E-06	4.65E-06	0.000109302	0	0	8.62E-06
Ruminococcaceae	0.001088372	0.003290244	0.002439535	0.002506977	0.002376744	0.002792105	0.00322069
Ruminococcaceae (CAG-352)	0.000153488	0.000521951	2.33E-05	0.000430233	3.72E-05	0.000136842	0.000163793
Ruminococcaceae (UCG-001)	0.000504651	0.00112439	0.001797674	0.000718605	0.001130233	0.002573684	0.002834483
Ruminococcus	0.024148837	0.025739024	0.019334884	0.030853488	0.021474419	0.027018421	0.024784483
Saccharofermentans	0.000297674	0.002092683	0.000897674	0.000927907	0.002406977	0.002171053	0.001948276
Sanguibacter-Flavimobilis	5.12E-05	0	0	0	0	0	1.55E-05
Schwartzia	0.000167442	0	0	0	0	0	0
Secundilactobacillus	0	4.88E-06	0	4.19E-05	0	2.63E-06	6.90E-06
Selenomonas	4.65E-05	5.12E-05	0	0.000290698	2.33E-06	2.63E-06	6.90E-06
Serratia	4.65E-06	1.22E-05	3.02E-05	0	0	0	5.17E-06
Shuttleworthia	0.001318605	0.00222439	0.002083721	0.002562791	0.002267442	0.001826316	0.003896552
Slackia	0.002055814	0.001578049	0.002393023	0.001090698	0.0034	0.003276316	0.004105172
Solobacterium	0.002253488	0.001304878	0.000562791	0.005162791	0.000825581	0.001242105	0.000998276
Sphaerochaeta	0	4.15E-05	1.40E-05	0.000209302	1.16E-05	5.26E-06	2.07E-05

Sphingomonas	2.33E-06	2.44E-06	2.33E-06	2.33E-06	2.79E-05	1.05E-05	2.24E-05
Staphylococcus	2.09E-05	8.54E-05	0	2.79E-05	7.21E-05	1.05E-05	7.59E-05
Stenotrophomonas	0.000283721	3.90E-05	2.09E-05	2.56E-05	2.33E-06	1.05E-05	2.59E-05
Streptococcus	0.006632558	0.003770732	0.005846512	0.012888372	0.00457907	0.002534211	0.004682759
Succiniclasticum	9.77E-05	0.00032439	0.000234884	0.000181395	0.000225581	8.95E-05	2.24E-05
Succinivibrionaceae UCG-002	5.81E-05	2.44E-06	3.72E-05	6.51E-05	2.56E-05	1.32E-05	1.72E-06
Sulfuritalea	0.000281395	0	7.67E-05	0	6.98E-05	0	3.45E-06
Suttonella	0	0.000129268	0	1.40E-05	9.53E-05	0	0.000127586
Syntrophococcus	0.007313953	0.005485366	0.008711628	0.011488372	0.005793023	0.005313158	0.006196552
Tannerella	0.001065116	0.000431707	5.35E-05	9.53E-05	0.00035814	0.000118421	0.00015
Terrimonas	4.65E-06	1.71E-05	0	6.98E-06	1.86E-05	0	3.45E-05
Treponema	0.015690698	0.0338	0.005948837	0.01245814	0.009890698	0.026528947	0.023068966
Turicibacter	4.88E-05	0	0	1.16E-05	4.65E-06	7.89E-06	0
Tyzzerella	0.013413953	0.007258537	0.000251163	0.000227907	0.000481395	0.000494737	0.000210345
U29-B03	0.000393023	0.001821951	0.00255814	0.001455814	0.001655814	0.000978947	0.001025862
Unclassified Actinomycetaceae	9.53E-05	0.000131707	0.000227907	8.84E-05	0.000251163	1.58E-05	0.000184483
Unclassified Anaerovoracaceae	0.003069767	0.003429268	0.003625581	0.001911628	0.00515814	0.005560526	0.004255172
Unclassified Atopobiaceae	0.002130233	0.001182927	0.004690698	0.002346512	0.002704651	0.003965789	0.004460345
Unclassified Bacilli	2.56E-05	2.93E-05	5.35E-05	2.33E-05	6.05E-05	1.05E-05	8.79E-05
Unclassified Bacteroidales	0.004276744	0.00354878	0.001530233	0.001776744	0.007255814	0.016418421	0.005498276
Unclassified Beijerinckiaceae	1.86E-05	6.10E-05	7.67E-05	4.88E-05	6.28E-05	5.00E-05	0.000155172
Unclassified Burkholderiales	2.09E-05	0.000229268	0.000225581	5.58E-05	0.000162791	9.74E-05	0.000298276
Unclassified Cardiobacteriaceae	0.000134884	2.93E-05	0	6.28E-05	0	0	0.000194828
Unclassified Clostridia	0.014360465	0.00844878	0.008232558	0.014165116	0.008934884	0.011578947	0.014324138
Unclassified Coriobacteriales	0.0048	0.002463415	0.005560465	0.005632558	0.003895349	0.003163158	0.006713793
Unclassified Coriobacteriales Incertae Sedis	0.003609302	0.002170732	0.00224186	0.001986047	0.003186047	0.002668421	0.001851724
Unclassified Eggerthellaceae	0.002955814	0.004346341	0.00692093	0.004423256	0.006588372	0.0059	0.010444828
Unclassified Erysipelatoclostridiaceae	0.000295349	0.001531707	0.001388372	0.000504651	0.00015814	3.95E-05	0.000337931

Unclassified Erysipelotrichaceae	0.000367442	0.001217073	0.000162791	0.000606977	0.000404651	0.000178947	0.000236207
Unclassified Firmicutes	0.005262791	0.003282927	0.00085814	0.001346512	0.000867442	0.001734211	0.001155172
Unclassified Gammaproteobacteria	0.000155814	0.00054878	0.000388372	0.000262791	0.000611628	0.000371053	0.000698276
Unclassified Lachnospiraceae	0.040948837	0.036612195	0.03745814	0.033790698	0.03964186	0.064568421	0.061906897
Unclassified Lactobacillaceae	0.00024186	0.000212195	8.84E-05	0.00054186	0.00024186	0.000318421	0.000393103
Unclassified Microbacteriaceae	4.65E-06	9.76E-06	2.79E-05	4.65E-06	4.65E-06	1.58E-05	0.00012069
Unclassified Negativicutes	0.000239535	0.002034146	0.000169767	8.37E-05	0.001586047	0.000810526	0.00042931
Unclassified Oscillospiraceae	0.004237209	0.003114634	0.002234884	0.001167442	0.002613953	0.005389474	0.00525
Unclassified Oscillospirales	0.004597674	0.002860976	0.002327907	0.001276744	0.001744186	0.001684211	0.001967241
Unclassified Pasteurellaceae	2.33E-06	0	8.37E-05	2.33E-06	0	0	0
Unclassified Peptostreptococcaceae	8.60E-05	0	0	1.63E-05	0	5.00E-05	0
Unclassified Prevotellaceae	0.015737209	0.011590244	0.008383721	0.006230233	0.006027907	0.009913158	0.007612069
Unclassified Proteobacteria	1.40E-05	2.44E-06	0	0	4.65E-06	0	1.72E-06
Unclassified Ruminococcaceae	0.00084186	0.001956098	0.003462791	0.000706977	0.000827907	0.003478947	0.00167069
Unclassified Selenomonadaceae	0.001186047	0.000219512	0.000337209	9.30E-05	0.001011628	0.000178947	0.000332759
Unclassified Sporomusaceae	0	0	0	0	0	2.37E-05	8.62E-06
Unclassified Veillonellaceae	1.40E-05	0	0	0.000313953	0	1.05E-05	3.45E-06
Unclassified Veillonellales-Selenomonadales	0.008986047	0.003187805	0.000744186	0.001632558	0.002253488	0.0031	0.00327931
Unclassified Xanthobacteraceae	0	2.44E-06	6.98E-06	0	2.33E-06	0	1.72E-05
Uncultured Actinomycetaceae	0.000302326	0.000136585	0.000134884	1.63E-05	0.000167442	0.000381579	0.000165517
Uncultured Atopobiaceae	0.004504651	0.003104878	0.003560465	0.003488372	0.003462791	0.002594737	0.003803448
Uncultured Bacteroidales	0.000390698	0.001078049	0.000451163	0.000623256	0.00035814	0.000565789	0.000308621
Uncultured Beggiatoaceae	0.000248837	0	6.98E-05	0	0.000130233	0	5.17E-06
Uncultured Beijerinckiaceae	0	2.44E-06	4.65E-06	0	0	7.89E-06	1.38E-05
Uncultured Coriobacteriales	9.07E-05	8.29E-05	0.000123256	7.21E-05	9.30E-05	0.000173684	0.00037069
Uncultured Coriobacteriales_Incertae_Sedis	0.00125814	0.000221951	6.74E-05	0.001002326	0.000139535	7.37E-05	0.000143103
Uncultured Dysgonomonadaceae	4.65E-06	7.32E-06	5.58E-05	0	5.58E-05	1.05E-05	5.17E-06
Uncultured Eggerthellaceae	0.000386047	0.000253659	0.000832558	0.000932558	0.000409302	0.000244737	0.000491379

Uncultured Erysipelotrichaceae	0.001530233	0.001395122	0.000667442	0.001725581	0.001090698	0.001123684	0.000758621
Uncultured Lachnospiraceae	0.019155814	0.022314634	0.029283721	0.016293023	0.01582093	0.014981579	0.018713793
Uncultured Marinifilaceae	0.000165116	0.00037561	2.33E-06	2.00E-04	0.000134884	9.47E-05	5.00E-05
Uncultured Oscillospiraceae	0.000493023	0.002446341	0.000430233	0.000260465	0.001832558	0.005694737	0.00337069
Uncultured Paracaedibacteraceae	0.000583721	0.000378049	0.000562791	0.00075814	0.000706977	0.001113158	0.001193103
Uncultured Peptococcaceae	4.88E-05	0.000165854	0.000102326	3.49E-05	2.56E-05	1.84E-05	1.90E-05
Uncultured Peptostreptococcaceae	1.40E-05	0	0	0	3.02E-05	0	2.24E-05
Uncultured Prevotellaceae	0	2.20E-05	2.33E-06	0	0	0	1.38E-05
Uncultured Rhodospirillales	3.49E-05	3.90E-05	4.19E-05	2.33E-05	3.49E-05	7.89E-05	3.62E-05
Uncultured Rickettsiales	1.40E-05	1.22E-05	2.33E-05	0.000123256	0.000127907	1.58E-05	6.90E-06
Uncultured Ruminococcaceae	0.005113953	0.00194878	0.001144186	0.001986047	0.002976744	0.0019	0.000782759
Uncultured Selenomonadaceae	0.001060465	0.000482927	0.000109302	0.000753488	0.000246512	7.37E-05	9.48E-05
Uncultured Succinivibrionaceae	0.000260465	0.000163415	0	0.000169767	1.00E-04	2.37E-05	5.17E-05
Veillonellaceae UCG-001	6.74E-05	1.71E-05	7.21E-05	0.000293023	1.86E-05	6.84E-05	3.28E-05
WCHB1-41	2.79E-05	6.10E-05	0.000444186	2.56E-05	9.07E-05	4.47E-05	4.14E-05
Weissella	0	0.000121951	0.005097674	2.56E-05	2.33E-06	2.37E-05	3.79E-05
[Anaerorhabdus] Furcosa Group	6.51E-05	0.000165854	9.07E-05	7.44E-05	0.000169767	6.05E-05	6.72E-05
[Clostridium] Methylpentosum Group	0.002132558	0.000131707	0	8.37E-05	2.33E-06	6.58E-05	1.21E-05
[Eubacterium] Brachy Group	0.00017907	0.00042439	0.000186047	0.000118605	0.000165116	0.000168421	0.00015
[Eubacterium] Hallii Group	0.004204651	0.004046341	0.006183721	0.004355814	0.009132558	0.010505263	0.009337931
[Eubacterium] Nodatum Group	0.006483721	0.005395122	0.006734884	0.005288372	0.010132558	0.008286842	0.0066
[Eubacterium] Ruminantium Group	0.000765116	0.002126829	0.001362791	0.000506977	0.003060465	0.002486842	0.001660345
[Eubacterium] Saphenum Group	4.88E-05	0	2.33E-06	2.09E-05	1.86E-05	5.79E-05	2.76E-05
[Eubacterium] Siraeum Group	0	0	0	0	0	1.84E-05	4.31E-05
[Eubacterium] Ventriosum Group	0.00115814	0.000339024	0.001044186	8.60E-05	0.000460465	0.000678947	0.001365517
[Eubacterium] Xylanophilum Group	0.00014186	0.000556098	0.000981395	0.000327907	8.00E-04	0.0015	0.003860345
[Ruminococcus] Gauvreauii Group	0.002223256	0.001831707	0.001746512	0.001353488	0.0011	0.000289474	0.000436207
[Ruminococcus] Torques Group	2.33E-05	5.61E-05	5.81E-05	9.07E-05	7.91E-05	0.000155263	0.000113793

Table S 14 Significant differences in the relative abundance of the 43 top genera of all habitats. Selection of genera based on the genera shown in Fig. S 3. The Kruskal-Wallis test with Bonferroni correction was applied. The p-values given represent the asymptotic significance (2-tailed test). < means that the corresponding genus has a significantly lower abundance in this habitat than in the corresponding habitat listed. Members of the core microbiota are marked [core]

Genus	AG	ABF	BF	PF	SF	GSF	AMF
Prevotella [Core]			< AG 0.012,<br>< ABF 0.001,<br>< SF 0.002,<br>< GSF 0.007				
Eggerthellaceae (Dnf00809) [Core]	< PF 0.015,<br>< AMF 0.001	< BF 0.002,<br>< PF 0.000,<br>< SF 0.001,<br>< GSF 0.001,<br>< AMF 0.000					
Unclassified Lachnospiraceae [Core]	< GSF 0.002,<br>< AMF 0.002	< GSF 0.000,<br>< AMF 0.000	< GSF 0.003,<br>< AMF 0.003	< GSF 0.000,<br>< AMF 0.000			
Quinella			< AG 0.000,<br>< ABF 0.000,<br>< PF 0.001,<br>< AMF 0.014		< AG 0.030,<br>< ABF 0.001	< ABF 0.007	< ABF 0.003
Oscillospiraceae (NK4A214) [Core]	< ABF 0.001,<br>< BF 0.000,<br>< PF 0.014,<br>< SF 0.007,<br>< GSF 0.000,<br>< AMF 0.000						
Ruminococcus	< GSF 0.034						
Olsenella		< BF 0.004,<br>< PF 0.030				< BF 0.004,<br>< PF 0.028	
Clostridia (UCG-014)	< BF 0.000,<br>< SF 0.000,<br>< GSF 0.000,<br>< AMF 0.000	< BF 0.002,<br>< SF 0.013		< BF 0.000,<br>< SF 0.000,<br>< GSF 0.000,<br>< AMF 0.001			
Candidatus Saccharimonas	< AMF 0.005	< BF 0.008,<br>< SF 0.000,<br>< GSF 0.000,<br>< AMF 0.000		< SF 0.014,<br>< GSF 0.000,<br>< AMF 0.000			
Uncultured Lachnospiraceae						< BF 0.049	
Treponema	< ABF 0.006		< ABF 0.000,<br>< GSF 0.000,<br>< AMF 0.000	< ABF 0.001	< ABF 0.000,<br>< GSF 0.036,<br>< AMF 0.012		
Prevotellaceae (UCG001)			< AG 0.044,<br>< ABF 0.002,<br>< SF 0.001,<br>< GSF 0.030	< ABF 0.050,<br>< SF 0.018			

Lachnospiraceae (NK3A20)			< AG 0.003, < PF 0.000			< AG 0.008,<br>< PF 0.000	< AG 0.000,<br>< ABF 0.001,<br>< PF 0.000,<br>< SF 0.004
Bacilli (RF39)	< BF 0.000,<br>< SF 0.000,<br>< GSF 0.000,<br>< AMF 0.000	< BF 0.005,<br>< SF 0.000,<br>< GSF 0.000,<br>< AMF 0.000		< BF 0.000,<br>< SF 0.000,<br>< GSF 0.000,<br>< AMF 0.000			
Oscillospirales (UCG011)	< BF 0.000,<br>< SF 0.010,<br>< GSF 0.000,<br>< AMF 0.000	< BF 0.000,<br>< SF 0.008,<br>< GSF 0.000,<br>< AMF 0.000					
Eubacterium coprostanoligenes group						< PF 0.006	
Unclassified Clostridia		< AMF 0.048					
Marvinbryantia	< AMF 0.010	< AMF 0.024		< GSF 0.005,<br>< AMF 0.000			
Unclassified Prevotellaceae	< ABF 0.010,<br>< GSF 0.000,<br>< AMF 0.009			< GSF 0.017			
Butyrivibrio	< SF 0.000,<br>< GSF 0.000,<br>< AMF 0.014	< GSF 0.044	< GSF 0.008	< SF 0.008,<br>< GSF 0.000			< GSF 0.035
Enterorhabdus	< AMF 0.000	< BF 0.004,<br>< SF 0.024,<br>< GSF 0.019,<br>< AMF 0.000		< AMF 0.001			
Muribaculaceae			< ABF 0.000,<br>< AMF 0.014				
Fretibacterium			< AG 0.026,<br>< ABF 0.000		< ABF 0.042		
Latilactobacillus	< BF 0.005	< BF 0.001			< BF 0.000	< BF 0.000	< BF 0.008
Prevotellaceae (UCG003)			< ABF 0.003,<br>< SF 0.000,<br>< AMF 0.003				
Eubacterium nodatum group				< SF 0.004,<br>< GSF 0.045,<br>< AMF 0.031			
Eubacterium hallii group	< SF 0.000,<br>< GSF 0.000,<br>< AMF 0.032	< SF 0.006,<br>< GSF 0.000	< GSF 0.011	< SF 0.006,<br>< GSF 0.000			< GSF 0.029

Unclassified Eggerthellaceae	< BF 0.000,<br>< SF 0.000,<br>< GSF 0.004,<br>< AMF 0.000	< BF 0.023,<br>< AMF 0.000		< BF 0.031,<br>< AMF 0.000			
Unclassified Bacteroidales	< SF 0.014,<br>< GSF 0.000,<br>< AMF 0.000	< GSF 0.000,<br>< AMF 0.035	< SF 0.008,<br>< GSF 0.000,<br>< AMF 0.000	< SF 0.004,<br>< GSF 0.000,<br>< AMF 0.000			
Ralstonia	< ABF 0.000,<br>< PF 0.007,<br>< GSF 0.000,<br>< AMF 0.000		< ABF 0.001,<br>< AMF 0.001		< ABF 0.000,<br>< PF 0.000,<br>< GSF 0.000,<br>< AMF 0.000		
Tyzzerella				< ABF 0.044	< ABF 0.025		
Enterococcus	< BF 0.021	< BF 0.000			< BF 0.000		< BF 0.007
Leuconostoc	< BF 0.015				< BF 0.004		< BF 0.002

References

1. Onderscheka K and Jordan HR (1976) *Einfluß der Jahreszeit, des Biotops und der Äsungskonkurrenz auf die botanische Zusammensetzung des Panseninhaltes beim Gams-, Reh-, Muffel- und Rotwild.* Die Bodenkultur. **27**(2):202-217
2. König A, Scheingraber M, and Mitschke J, *Energiegehalt und Qualität der Nahrung von Rehen (Capreolus capreolus) im Jahresverlauf in zwei unterschiedlich geprägten Habitaten*. Forstliche Forschungsberichte. Vol. 215. 2016, Freising: Zentrum Wald-Forst-Holz
3. Kölling C, Dalhäuser H, Foerst K, Kreutzer K, Sammler P, and Ewald J, *Waldatlas Bayern*, in *Forstliche Wuchsgebiete und Wuchsbezirke in der Bundesrepublik Deutschland*. 2005, Bayerische Landesanstalt für Wald und Forstwirtschaft: Freising
